# Tree defenses, host choice, and reproductive success of a native bark beetle under novel outbreak conditions

**DOI:** 10.1002/eap.70176

**Published:** 2026-01-14

**Authors:** Grace Graham, Marcella Windmuller‐Campione, Daniel Griffin, Fraser McKee, Brian Aukema

**Affiliations:** ^1^ Department of Entomology University of Minnesota St. Paul Minnesota USA; ^2^ Department of Forest Resources University of Minnesota St. Paul Minnesota USA; ^3^ Department of Geography, Environment & Society University of Minnesota Minneapolis Minnesota USA; ^4^ Saint Anthony Falls Laboratory University of Minnesota Minneapolis Minnesota USA; ^5^ Present address: Alaska Division of Forestry and Fire Protection Anchorage Alaska USA; ^6^ Present address: Alberta Forestry & Parks Rocky Mountain House Alberta Canada

**Keywords:** bark beetles, climate change, coevolution, *Dendroctonus simplex*, eastern larch, eastern larch beetle, forest health, *Larix laricina*, resin defenses, tamarack

## Abstract

Bark beetles of the genus *Dendroctonus* are some of the most important disturbance agents in North American forests, having colonized conifers for millions of years. The selection pressure posed by tree‐killing bark beetles pushed trees to develop an arsenal of defensive strategies to which beetles have adapted in their turn. Recent surges in bark beetle‐related tree mortality have highlighted the potential of novel climatic and landscape conditions to push tightly calibrated relationships beyond historical norms. One such example is an unprecedented outbreak of the native eastern larch beetle (ELB), *Dendroctonus simplex* LeConte (Coleoptera: Curculionidae; Scolytinae), that has killed eastern larch (tamarack), *Larix laricina* (Du Roi) K. Koch, trees across more than 460,000 ha of forest in the Great Lakes Region since 2001. The ability of a bark beetle to attack healthy trees is dependent on sufficient local beetle numbers to overwhelm host defenses and a behavioral switch to target those trees that are avoided at lower population levels. ELB was not previously considered an aggressive tree colonizer, but extended growing seasons have contributed to recent eruptions in local populations of the species. We combined a dendrochronological analysis of tree cores with observational data collected from 2011 to 2013 in Beltrami Island State Forest, Minnesota, to understand tree defensive capacity and beetle outbreak dynamics in this understudied system. We found that preformed defenses visible in tamarack xylem were limited and did not determine host preference of ELB during our study. Beetles colonized the largest trees with the thickest phloem regardless of defensive capacity. Preformed resin defenses measured in tree phloem were correlated with reduced beetle reproductive success but were unrelated to resin metrics from tree xylem. With this work, the interaction between ELB and tamarack serves as a model to explore how climate change may alter species associations within native forest systems and the management challenges associated with underestimating historically benign pests.

## INTRODUCTION

Bark beetles of the genus *Dendroctonus* (Latin for “tree killers”) have caused widespread mortality of trees across North America and are considered one of the most important disturbance agents in coniferous forests (Dodge, 1938; Hopkins, 1908; Raffa et al., 2008; Wood, 1963). Spending most of their lives under the bark of hosts, each *Dendroctonus* bark beetle species colonizes only one or a few closely related tree species (Hopkins, 1909; Wood, 1963). A large number of beetles feeding in one tree can kill a host by introducing pathogenic fungi, destroying the phloem, and cutting off nutrient and water transport systems (Berryman, 1972; Hopkins, 1908; Raffa & Berryman, 1983). Devastating outbreaks can persist until the availability of host trees is exhausted or other, often unknown, factors help diminish beetle populations (Biedermann et al., 2019; Raffa et al., 2008). Given their economic and ecological significance, over 100 years of western scientific scholarship have been devoted to bark beetles, their complex relationship with host trees, and how management decisions might contribute to productive forests (Aukema et al., 2016; Biedermann et al., 2019; Fettig et al., 2007; Hopkins, 1908; Windmuller‐Campione et al., 2021; Wood, 1982). Previous research, however, generally focused on controlling outbreaking populations to limit financial losses, leaving many gaps regarding beetle species that attack less commercially important trees and beetle populations that do not regularly exhibit aggressive behavior (Aukema et al., 2016; Hopkins, 1908).

The tree‐killing reputation of the *Dendroctonus* genus is based on observations of epidemic outbreaks in which rapidly building populations of beetles attack healthy trees (Hopkins, 1908; Raffa et al., 2008; Wood, 1982). However, the majority of bark beetle populations feed upon and reproduce in cut logs or trees stressed by old age, fungal infection, injuries, or drought (Dodge, 1938; Hopkins, 1908; Raffa & Berryman, 1983; Wood, 1982). Usually there is a limited supply of such trees, and bark beetle populations are maintained at endemic levels. Endemic populations of bark beetles contribute to the regular reduction in stand density and promotion of stand heterogeneity found in healthy forests (Bentz, 2008; Christiansen et al., 1987; Raffa et al., 2008). Sporadic outbreaks are possible for many bark beetle species. Such events are often temporary and localized to specific landscape‐level disturbances that increase the amount of weakened or dead material available to beetles. Fire, defoliation, drought, windstorms, and improper slash management during logging operations have all contributed to previously recorded bark beetle outbreaks (Dodge, 1938; Wood, 1982). Historically, forests have been resilient to such increases in beetle pressure, and traditional preventative management approaches may succeed in controlling these types of outbreaks (Fettig et al., 2007, 2022; Mattson, 1996; Raffa et al., 2009; Windmuller‐Campione et al., 2021).

Anthropogenic factors such as climate change related increases in temperature and drought, and the legacy of historic management focused on single‐species, even‐aged stands have created more vulnerable landscapes and contributed to recent escalation in the number and severity of bark beetle outbreaks (Bentz, 2008; Logan et al., 2003; Raffa et al., 2008). Included in this trend are large‐scale eruptions of species not typically considered to be major forest health threats (Logan et al., 2003; Raffa et al., 2008). An example is the unprecedented outbreak of eastern larch beetle (ELB), *Dendroctonus simplex* LeConte (Coleoptera: Curculionidae; Scolytinae) in the eastern larch (tamarack), *Larix laricina* (Du Roi) K. Koch forests of the Great Lakes Region (Aukema et al., 2016; Minnesota Department of Natural Resources, 2025).

One of the key determinants of beetle‐host dynamics is tree defenses—most bark beetles do not attack healthy trees because the defenses of live, vigorous specimens can ward off small numbers of attacking beetles (Krokene, 2015; Raffa et al., 2005; Raffa & Berryman, 1983). Over 45 million years of coevolution, conifers have developed a range of physical and chemical structures to deter beetle entry, aggregation, and brood establishment and fight infection from beetle‐associated fungi (Brunelle et al., 2008; Krokene, 2015; Labandeira et al., 2001; Raffa & Berryman, 1983). When contending with aggressive bark beetle species, such defenses are most effective at low beetle population levels. When beetle populations exceed the threshold necessary to overwhelm the defenses of vigorous trees, extensive outbreaks can occur (Boone et al., 2011; Raffa & Berryman, 1983). Host choice is predicated on beetle population levels, and the preference for more vigorous hosts represents a behavioral flip from individuals seeking the less risky, lower quality reproductive substrate in poorly defended trees to favoring the high risk, high reward of entering large, heavily fortified trees (Boone et al., 2011; Raffa & Berryman, 1983; Wallin & Raffa, 2004). However, after outbreaks, some large, living trees persist on the landscape. Understanding what sets those trees that survive an outbreak apart from those that do not is of interest to both researchers and forest managers (Biedermann et al., 2019; Ferrenberg et al., 2014; Hood et al., 2015; Nebeker et al., 1992; Raffa & Berryman, 1982; Strom et al., 2002). Additionally, even when protections are breached during beetle epidemics, individual tree defenses may continue to influence pest fitness within the stand by reducing reproductive success, but little research has been done in this realm (Berryman, 1976; Biedermann et al., 2019; Clark et al., 2012; Lieutier, 2002).

The primary defensive strategy for members of *Pinaceae* is the synthesis and exudation of resin, a viscous compound stored under pressure that contains various chemicals used to neutralize fungal or insect threats and heal wounds (Franceschi et al., 2005; Krokene, 2015; Raffa & Berryman, 1983). Axial resin ducts running vertically within the secondary xylem of the tree bole include epithelial cells that synthesize resin and an intercellular canal. These features store resin, help maintain exudation pressure, and serve as initiation points for the radial ducts that transport resin from the inner wood to the bark (Franceschi et al., 2005; Krokene, 2015; Larson, 1994). In addition to these interconnected ducts, the phloem also contains its own resin storage and synthesis structures known as resin blisters and cells (Franceschi et al., 2005; Krokene, 2015). Produced initially via cell division and differentiation in the vascular cambium, some of these structures are constitutive, that is, built as a normal tree process regardless of direct experiences with threats. Others, including structures known as traumatic resin ducts, are induced, formed, or activated in response to injury (Berryman, 1972; Franceschi et al., 2005; Thomson & Sifton, 1926). The combination of latent defenses and responsive upregulation allows a tree to be flexible in addressing dangers without divesting too many resources from growth or other needs (Franceschi et al., 2005; Krokene, 2015; Lombardero et al., 2000; Raffa et al., 2005).

Many research groups have examined the role of resin defenses in determining inter and intraspecific tree resistance to bark beetles at various population levels, but methodologies have been diverse and trends have been difficult to parse. Some concluded that measures of constitutive resin quantity could be an effective tool to determine more resistant individuals or genetic stock (Nebeker et al., 1992; Strom et al., 2002; Tisdale et al., 2003). Others determined that the rate or size of the induced response was a more important factor (DeRose et al., 2017; Raffa & Berryman, 1982; Schiebe et al., 2012). Yet another group found that which resin measures matter depended on local beetle populations, and, under high beetle pressure, no measured defensive trait predicted tree survival (Boone et al., 2011). Since axial resin ducts are embedded within the radial file of xylem tracheids of annual growth rings, they can be dated to specific events and serve as a surrogate for understanding a tree's investment in defenses over the course of its lifetime (Hood & Sala, 2015; Vázquez‐González et al., 2020). As such, there has been increased utilization of dendrochronological analysis of axial resin duct components in the xylem as a more straightforward proxy for assessing resin defenses (Bentz et al., 2016; Blanche et al., 1992; Ferrenberg et al., 2014; Hodges et al., 1981; Hood et al., 2015; Kane & Kolb, 2010; Lombardero et al., 2000; Mason et al., 2019; Yi et al., 2021; Zhao et al., 2019; Zhao & Erbilgin, 2019). Even within this narrower realm of tree defense research, the significance of specific metrics for tree resistance has not been conclusively established (Hood & Sala, 2015; Vázquez‐González et al., 2020).

Nevertheless, given the relative simplicity of acquiring tree cores and the regular collection and storage of these specimens within forestry research (Stokes & Smiley, 1996), such approaches may open exciting avenues for exploring tree defenses in bark beetle systems. Technological advancements in the visualization and measurement of tree cores (Griffin et al., 2021; Hood et al., 2020) also allow for the more consistent quantification of axial resin features called for by researchers (Vázquez‐González et al., 2020). However, these methods have not been applied extensively outside the genus *Pinus* and have never been used in tamarack. Our goal is to apply these new dendrochronological tools to visualize and measure resin duct features in an archived collection of tamarack wood cores acquired during previous field work tracking an outbreak of ELB. With this approach, we hope to examine beetle population dynamics and tree defenses in a historically understudied bark beetle system. To that end, we ask: (1) What is the defensive capacity of tamarack visible in tree cores? (2) How do tamarack defenses influence ELB host selection and reproductive success? (3) What factors best predict ELB colonization patterns under novel outbreak conditions? Answering these questions will help substantiate an integrated methodology for studying resin‐based conifer defenses and uncover factors shaping the outbreak dynamics of bark beetles under changing climatic conditions.

## METHODS

### Study system

Located near the Canadian border in north‐central Minnesota, Beltrami Island State Forest falls within the Agassiz Lowlands subsection of the Laurentian Mixed Forest ecological province (212 Mb) as defined by the state of Minnesota (Hanson & Hargrave, 1996; North, 2013). The climate features a short, mild growing season and harsh winters with average temperatures ranging between −20.7°C in January and 20.6°C in July and typical total annual precipitation around 636.3 mm (Palecki et al., 2021). Dominant plant communities include lowland conifer stands, upland aspen forests, and non‐forested sedge wetlands. Current forest conditions were shaped by the boreal climate, glacial soil deposition patterns, and a disturbance history that includes shifting fire regimes, settlement era ditching and draining, and varying management intensities of tree harvest and planting (North, 2013).

Minnesota lies along the southern margin of tamarack distribution in North America, but the species is abundant across Beltrami Island State Forest, as it is tolerant of the low nutrient, poorly drained soil conditions common in a landscape formed by the geologic processes of retreating continental glaciers and lakebed depositions during the last ice age (Griffin, 1977; Johnston, 1990; Minnesota Department of Natural Resources, 2013; North, 2013). Once considered suitable for timber, the combination of extractive logging practices, outbreaks of defoliators in the early 1900s, and difficulties of harvesting in boggy terrain have limited the economic potential of tamarack in northern Minnesota (Drooz, 1960; Minnesota Department of Natural Resources, 2013). Consequently, this forest type is commonly under a passive management strategy (Minnesota Department of Natural Resources, 2013).

Recent interest in tamarack has been spurred by an unprecedented outbreak of ELB. This native insect has now damaged extensive regions of tamarack cover type in the Great Lakes Region of Canada and the United States, including over 460,000 ha of forest in Minnesota (Minnesota Department of Natural Resources, 2025). ELB can be found throughout the range of its primary host, but typical populations persist at endemic levels and subsist solely on recently dead or weakened host trees (Langor & Raske, 1989; Wood, 1982). Historically, ELB has been described as univoltine (Dodge, 1938). The most thorough description of a life cycle comes from a Newfoundland population (Langor & Raske, 1987), where pioneer female beetles emerged in spring, initiated attacks, and recruited male and additional female beetles to suitable host material within days. Monogamous pairs constructed egg galleries and established a first brood over the course of a month before reemerging and seeking additional, often poorer quality, host material for a sister brood. Beetles from the first brood emerged in late summer and early fall and walked down the bole to overwinter, but no beetles from the second brood emerged (Langor & Raske, 1987). Due to the wide distribution of ELB populations and the temperature‐dependent development of beetles, the more specific phenology and number of broods established vary both geographically and annually based on local host availability and climatic conditions (Mckee et al., 2022).

Although sporadic small‐scale outbreaks of ELB had been observed historically throughout its range, larger scale outbreaks in the 1970s in Alaska and eastern Canada revealed more aggressive behavior including mass attacks of healthy trees (Dodge, 1938; Langor & Raske, 1989). First observed in 2001, the current outbreak in Minnesota stands out from others due to its duration, absence of clear predisposing factors, impacts across varying site conditions, and lack of historical records of such an epidemic in local beetle populations (Langor & Raske, 1989; McKee et al., 2022; Minnesota Department of Natural Resources, 2013; Ward & Aukema, 2019). An additional summer generation and greater beetle survival in response to expanded growing seasons and climate warming have likely allowed beetle populations to grow to epidemic levels in the state (McKee et al., 2022; Venette & Walter, 2012). The Beltrami Island region has been an epicenter of activity for ELB throughout the outbreak and landscape‐level waves of tree mortality were evident during the initial study period from 2011 to 2013 (McKee, 2015; Minnesota Department of Natural Resources, 2012).

### Original field work

In 2011, we established three research sites in mature tamarack‐dominated stands along 9 km of Pitt Grade Road, maintaining a distance of at least 2.4 km between sites. All sites are contained within Beltrami Island State Forest in Lake of the Woods county, MN. We selected sites based on proximity to a known outbreak of ELB, abundance of large trees without infestations, and accessibility from maintained roads. Specific locations and the distribution of study trees among sites are summarized in Table 1. Following the first beetle flight in May 2011, we identified small epicenters of colonized trees surrounded by green, apparently healthy specimens within each of the sites. We randomly selected and assigned a unique identifier to 131 tamaracks with diameters larger than 10 cm for repeated observations.

**TABLE 1:** Characteristics of tamarack (*Larix laricina*) in three observational sites in northern Minnesota observed during an outbreak of eastern larch beetle (*Dendroctonus simplex*).

		Site		
Variable	Measure	1	2	3
GPS location (degrees)	lat/long	48.5997, −94.7577	48.5787, −94.7575	48.6537, −94.7583
Study trees	*n*	38	41	52
Trees colonized 2011	*n*	12	11	12
Trees colonized 2012	*n*	24	29	26
Trees colonized 2013	*n*	0	0	6
Trees escaped	*n*	2	1	8
dbh (cm)	Mean (SE)	17.7 (0.4)	18.4 (0.5)	17.6 (0.6)
Age (years)	Mean (SE)	49.9 (0.6)	41.5 (0.6)	37.5 (0.6)
Stand density (m^2^/ha)	Mean (SE)	18.8 (0.9)	22.1 (1.5)	12.4 (0.8)
Phloem thickness (mm)	Mean (SE)	2.5 (0.1)	3.2 (0.1)	3.1 (0.1)
Phloem resin cell density (no. cells/mm^2^)	Mean (SE)	7.3 (0.8)	6.9 (0.5)	8.5 (0.7)
Ring width 10 (mm/year)	Mean (SE)	1.11 (0.08)	2.06 (0.13)	1.88 (0.12)
BAI 10 (mm^2^/year)	Mean (SE)	706.80 (62.92)	1271.35 (95.85)	1089.42 (88.75)
Duct production 10 (no. ducts/year)	Mean (SE)	4.62 (0.39)	5.83 (0.31)	5.43 (0.32)
Duct size 10 (mm^2^)	Mean (SE)	0.007 (2.86E‐04)	0.007 (2.30E‐04)	0.007 (1.99E‐04)
Total duct area 10 (mm^2^/year)	Mean (SE)	0.032 (3.68E‐03)	0.041 (2.87E‐03)	0.037 (2.47E‐03)
Duct density 10 (no. ducts/mm^2^/year)	Mean (SE)	0.734 (0.08)	0.478 (0.05)	0.469 (0.04)
Relative duct area 10 (%)	Mean (SE)	0.47 (0.06)	0.31 (0.03)	0.31 (0.03)

*Note*: All trees characterized here were included in an initial observational study taking place from 2011 to 2013 in Beltrami Island State Forest, Minnesota. “Escaped” refers to those trees not colonized during the study period. A full description of variables can be found in Table 2. All statistics derived from individual core measurements were combined to the tree level and pooled across 10 years based on the year the tree was cored.

Abbreviations: BAI, basal area increment; dbh, diameter at breast height.

We monitored all trees for evidence of ELB attack (frass accumulation on the lower bole) every 7 days during the growing season in each of 2011, 2012, and 2013. Beetles had already attacked 28 study trees prior to the first observation point in June of 2011 and an additional 92 monitored trees were attacked throughout the multiyear study period. We randomly selected a subset of 52 from the pool of all colonized trees for additional assessment of beetle reproductive success. Once colonization was complete, we counted the number of attack points within the 16.5 × 30 cm (W × H) rectangle of bark that would later be covered by screen cages centered at 1.8 m above the ground on the north and south aspects of the bole. Collection cups affixed to the screen cages were checked twice weekly, and any adult beetles were counted and removed. Numbers of emerging brood adults were recorded throughout the summer and fall months and again the following spring to account for beetles that completed development over winter. Re‐emergent parent and brood adult beetles captured in screen cages were differentiated based on the timing of their emergence from trees compared to known phenological patterns (McKee, 2015). The count of brood adults was summed across the two screen cages, divided by the bark area covered by both cages (990 cm^2^), then divided by 9.9 to yield a total per 100 cm^2^ of infested bark. We calculated the number of brood adults per parent female using the number of attack points and a formula that accounts for the known rate of entrance hole sharing observed for ELB (Langor & Raske, 1987; McKee, 2015).

We measured tree characteristics including dbh, microsite stand density, and canopy class. Additionally, in the spring of each year of monitoring, we removed a 5 × 2 cm (H × W) rectangular phloem sample at 1.4 m above the ground from all non‐attacked trees for assessment of phloem thickness and resin cell density. For this work, we define “resin cells” as any large resin‐containing cavities visible in the phloem (Appendix S1: Figure S1). Our count may include other types of phloem resin reservoirs including resin blisters or enlarged resin ducts, but most structures were consistent in appearance with the “resin cells” described by Franceschi et al., 2005. We did not attempt to differentiate between the different types of phloem resin pools as all would be avoided by invading bark beetles (Berryman, 1972; Franceschi et al., 2005). The number of resin cells within phloem samples was counted on the same longitudinal section surface used for thickness measurements. Resin cell density was calculated by dividing this count by the cross‐sectional area (mean phloem thickness × 5 cm) of the sample. Further details regarding original field work methodologies can be found in supplemental materials (Appendix S1) and section A1.2 of McKee (2015).

### Dendrochronological analysis

Tree age, growth rate, and resin duct characteristics were analyzed using methods standard to tree ring research. During the original study, we chose two coring locations at heights of 20–25 cm and at least 90° apart for each tree based on likelihood of intercepting pith. We removed cores with a 5.15‐mm diameter increment borer (Haglöf Sweden AB, Langsele, Vasternorrland, Sverige, Sweden) in October of either the year of colonization if the tree was attacked or the final year of monitoring (2013) if the tree escaped attack. We measured tree diameter at coring height to the nearest 0.1 cm with dbh tape. Cores were originally mounted and sanded following standard techniques (Stokes & Smiley, 1996). We subsequently applied additional polishing with 15 and 9 μ microfinishing film (3M, St. Paul, MN, USA) as needed to optimize scrutiny of cellular anatomy.

**FIGURE 1:**
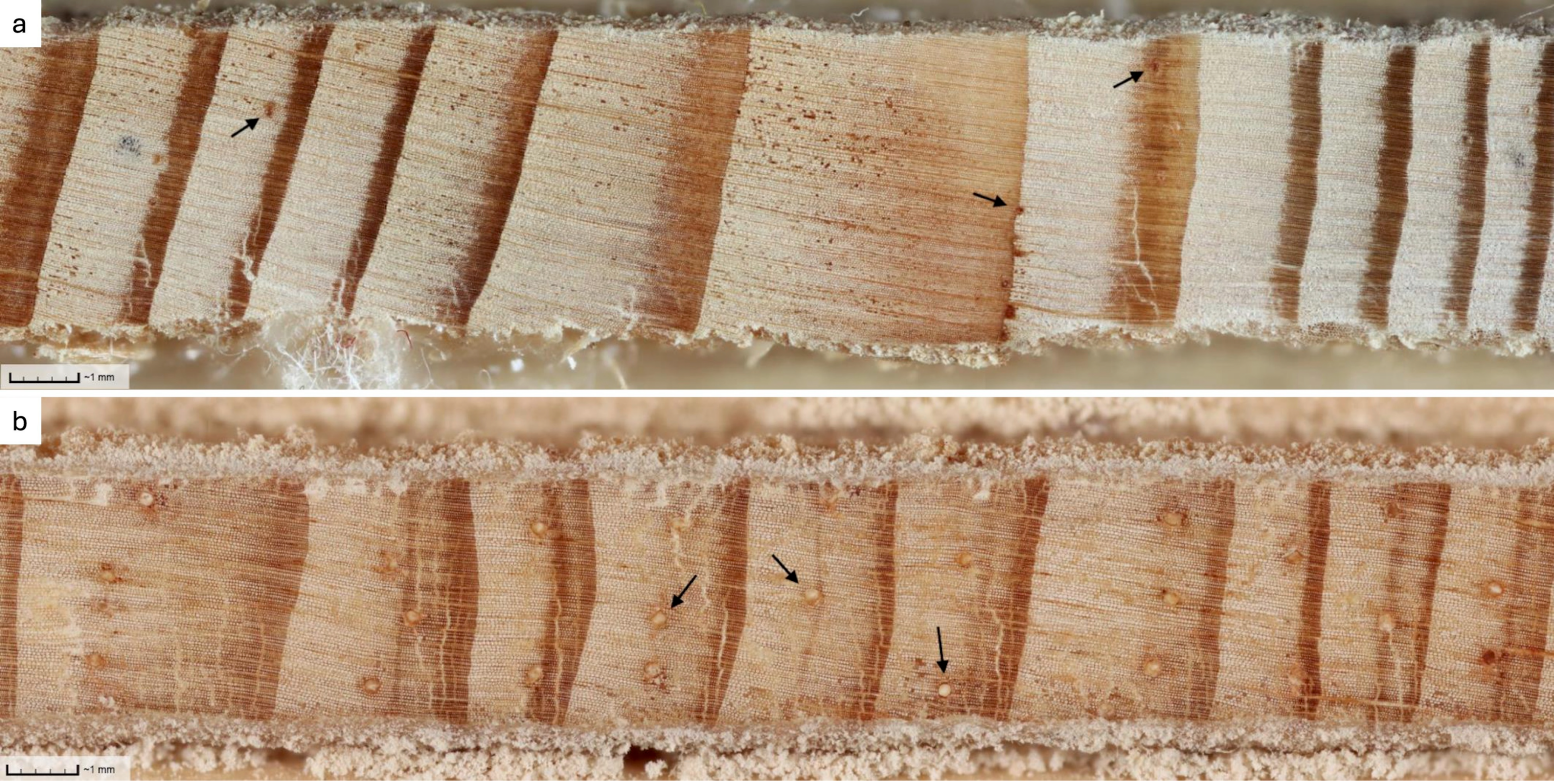
Examples of high‐resolution imagery of tree cores produced with our methodology and a comparison of the resin duct size and quantity visible in a subsection of cores from (a) tamarack (*Larix laricina*) and (b) ponderosa pine (*Pinus ponderosa*). Cores are scaled similarly and each display approximately 10 years of growth. Example resin ducts are indicated with black arrows. Pine core was collected in 2010 from a low elevation site in the Santa Catalina mountains near Tucson, Arizona, where the summer monsoon often creates an intra annual density fluctuation (Griffin, 2013). Tamarack core was collected in 2011 from Beltrami Island State Forest, Minnesota, during an outbreak of eastern larch beetle (*Dendroctonus simplex*). Both images are located on the DendroElevator (http://dendro.elevator.umn.edu) platform. Photo credit: (a) Grace Graham; (b) Daniel Griffin.

Using methodologies outlined in Griffin et al. (2021), we produced high‐resolution (~18,000 dpi) digital scans of tree cores (Figure 1) using a GigaMacro imaging system (Four Chambers Studio, LLC, Napa, CA, USA.) with subsequent focus‐stacking, mosaic‐stitching, and scale bar calibration using image editing programs developed by the team at the University of Minnesota AISOS imaging center. We uploaded images to DendroElevator (http://dendro.elevator.umn.edu), a platform that allows for online storage, visualization, and measurement of tree core scans. Ring widths representing annual growth were measured manually on images with micrometry tools available on the DendroElevator platform. Annual ring dates were initially estimated using the year of specimen collection as the datum in time. Visual cross‐dating of marker years between cores from the same tree and among trees from the same site was conducted to ensure proper assignment of calendar years to growth patterns and resin duct features, following methods standard in dendrochronology (Stokes & Smiley, 1996; Yamaguchi, 1991). Incomplete wood formation common in dying trees and at the base of trees where the cores were taken was a concern (Pallardy, 2008; Speer, 2010). We verified and, in some cases, adjusted calendar year dating of individual rings through use of dating quality control software COFECHA (Holmes, 1983), with settings of segment length set to 30 years, lag to 5 years, and cubic smoothing spline parameters maintained at 32 years.

Growth metrics included raw ring widths and a calculated basal area increment (BAI), which accounts for the proportion of the total core represented by a particular year's increment growth. We computed BAI from the raw ring widths and measured tree diameter at coring height, accounting for the phloem and bark by subtracting the average thickness of the phloem samples collected from individual trees at the beginning of each growing season. The calculations utilized the dplR package (v. 1.7.6) in R (Bunn, 2008; R Core Team, 2023). We estimated tree age by counting the number of rings to the pith or, when pith was not visible, using a stencil overlay on the core to calculate pith offset in a manner similar to the concentric circle method described by Applequist (1958) and Pirie et al. (2015).

We identified resin ducts based on their shape, presence of epithelial cells, and diagnostic compression of surrounding tracheid cells (Appendix S2: Figure S1; Pallardy, 2008; Hood et al., 2020). We included all ducts from the year 2001 onward, as this would capture at least 10 years of measurements for every core. Although cores were produced with a 5.15‐mm borer, inconsistencies in mounting and damage to visible areas prevented consistent measurement across the full width of all cores. To standardize the resin duct measurement area, we drew a 4‐mm wide visual guide across each core and only those ducts with centerpoints inside the set guidelines were recorded (Appendix S2: Figure S2). We manually drew an ellipse around each axial resin duct and its surrounding epithelial cells (Appendix S2: Figure S1b; Hood et al., 2020), measured the long and short axes of each ellipse and then estimated duct area using tools available in the DendroElevator platform. Each ellipse was tagged with the year and ring position in which it appeared (Appendix S2: Figure S3).

We calculated resin ducts metrics from raw ellipse measurements utilizing a workflow and R code adapted from Hood et al. (2020). These metrics include the mean size of all ducts per annual ring (“duct size”), the total number of ducts per annual ring (“duct production”), the sum of all duct areas per annual ring (“total duct area”), duct production divided by ring area (“duct density”), and the total duct area divided by ring area expressed as a percentage (“relative duct area”). The latter two are considered “standardized metrics” as they are adjusted based on annual ring area calculated as the ring width multiplied by the width of the core included for measurement (4 mm).

For tree‐level analysis, we averaged resin duct and growth metrics from paired cores following Hood et al. (2020), then pooled them in 5‐ and 10‐year increments based on the study year minus one to account for incomplete growth in those trees that were cored in the year of interest. For example, in 2011, 2012, and 2013 the 10‐year pool consisted of measurements from annual rings corresponding to 2001–2010, 2002–2011, and 2003–2012 respectively. Because not all sapwood continues to produce resin, this pooling scheme accounts for the recently formed resin duct infrastructure most likely to contribute to the actual resin volume beetles would encounter in the year of interest and aligns with other resin duct research (Ferrenberg et al., 2014; Hood & Sala, 2015; Kane & Kolb, 2010; Lewinsohn et al., 1991; Pallardy, 2008; Vázquez‐González et al., 2020). We did not attempt to differentiate between traumatic and constitutive resin ducts, as there is no consolidated methodology for such determinations across conifer species (Bollschweiler et al., 2008; Catherwood et al., 2022; DeRose et al., 2017; Gärtner & Heinrich, 2009; Nagy et al., 2000; Schneuwly et al., 2009; Wimmer & Grabner, 1997). All ducts, regardless of origin, would contribute to the overall resin capacity of the tree (Lewinsohn et al., 1991; Martin et al., 2002; Nebeker et al., 1992; Penhallow, 1907; Trapp & Croteau, 2001) and are considered “preformed” as, based on our pooling scheme, they existed prior to any beetle attacks in the year of interest. All tree characteristic variables including those determined from tree cores are summarized in Table 2.

**TABLE 2:** Summary of variables corresponding to tree characteristics.

Name	Description
dbh	Diameter at breast height (1.37 m) measured in cm
Age	Minimum estimate of tree age in years based on count of annual rings in tree cores
Stand density	A measure of microsite competition around an individual tree based on count of surrounding trees falling within 10 basal area factor (BAF) variable radius plots centered on tree of interest converted to meters^2^/hectare
Ring width 5/10	Annual increment width measured in mm/year averaged across the most recent 5 or 10 years of wood core
BAI 5/10	Basal area increment measured in mm^2^/year averaged across the most recent 5 or 10 years of wood core
Duct production 5/10	The total number of resin ducts per annual ring (no. ducts/year) averaged across the most recent 5 or 10 years of wood core (unstandardized)
Duct size 5/10	Mean area of resin ducts measured in mm^2^ in the most recent 5 or 10 years of annual rings of wood core (unstandardized)
Total duct area 5/10	Sum of resin duct area per annual ring measured as mm^2^/year averaged across the most recent 5 or 10 years of wood core (unstandardized)
Duct density 5/10	Total number of resin ducts per annual ring divided by the corresponding ring area (ring width × measured core width, i.e., 4 mm) expressed as no. ducts/mm^2^/year averaged across the most recent 5 or 10 years of wood core (standardized)
Relative duct area 5/10	Total resin duct area divided by annual ring area (ring width × measured core width, i.e., 4 mm) × 100 expressed as % averaged across the most recent 5 or 10 years of wood core (standardized)
Phloem thickness	Phloem thickness measured in mm of rectangular 5 × 2 sample removed from trees at the beginning of each summer averaged across three measurement points occurring at 0.5, 2.5, and 4.5 cm along the 5 cm length of sample
Phloem resin cell density	The number of resin cells counted across the same longitudinal section surface used for thickness measurements divided by the phloem cross‐sectional area (phloem thickness × length of sample, i.e., 5 mm) and expressed as number of cells/mm^2^

### Statistical analysis

We performed all analyses in R using the lme4 package (v1.1–35.1; Bates et al., 2015; R Core Team, 2023). When needed for linear mixed‐effects modeling, we generated test statistics for inferential tests such as ANOVA using degrees of freedom calculated via Satterthwaite's method within the lmerTest package (v3.1–3; Kuznetsova et al., 2017; R Core Team, 2023).

We examined relationships between the variables corresponding to tree characteristics (Table 2) and beetle dynamics via linear mixed‐effects models. As some traits were measured at multiple time points, those corresponding to the year of tree colonization were included in these models, and data collection year and site were included as non‐nested random effects. However, when examining colonization patterns across time, measurements from the year of interest were utilized for all trees. For this analysis, we categorized trees as colonized or not colonized for each year of the study based on field observations of beetle activity. Given the binary data distribution, we generated a separate generalized linear mixed‐effects model for the likelihood of a tree being colonized, with individual tree metrics as a fixed effect, site as a random effect, and a logit family link.

We examined *p*‐values of slope estimates and compared model Akaike information criterion (AIC) values to determine the best explanatory variable for a given response (Bozdogan, 1987). When necessary, we produced models with multiple fixed effects to compare combinations of explanatory variables to single variable models. We performed square root and log transformations for resin metrics and growth metrics respectively when necessary to satisfy assumptions of linear models, namely equal variance and normal distribution of residuals. Square root transformation was also required for the brood per tree and brood per female metrics. Due to the skewed nature of BAI values, a logarithmic transformation was performed on this variable throughout analysis.

We did not collect phloem from trees colonized prior to June 2011, so we could not include AIC values from phloem‐based metrics for comparisons for that year due to differing sample sizes compared to the other models. However, when phloem thickness or phloem resin cell density was a significant variable, we built multivariable models via forward selection to establish which variables exhibited the most explanatory power. After performing outlier tests, we removed one tree exhibiting several years of extremely small increment growth and one tree with highly skewed duct production from the analysis examining tree characteristics and resin traits. Similarly, we removed one small diameter, heavily attacked tree from beetle attack density models. Further note of these individuals is made in the results and discussion.

## RESULTS

Over the course of the original monitoring period, 92% of the study trees were colonized by ELB. Of those killed trees, most (66%) were attacked over the summer of 2012 (Table 1). By spring 2013, only 17 survivors remained, 11 of which escaped colonization entirely during the study. Attack densities on colonized trees ranged from 1.11 to 4.11 entry holes per 100 cm^2^ bark surface area with a mean of 2.16 attack points. An average of 10 brood adults per 100 cm^2^ successfully emerged from trees, which represents a mean of 3 brood adults per parent female.

**FIGURE 2:**
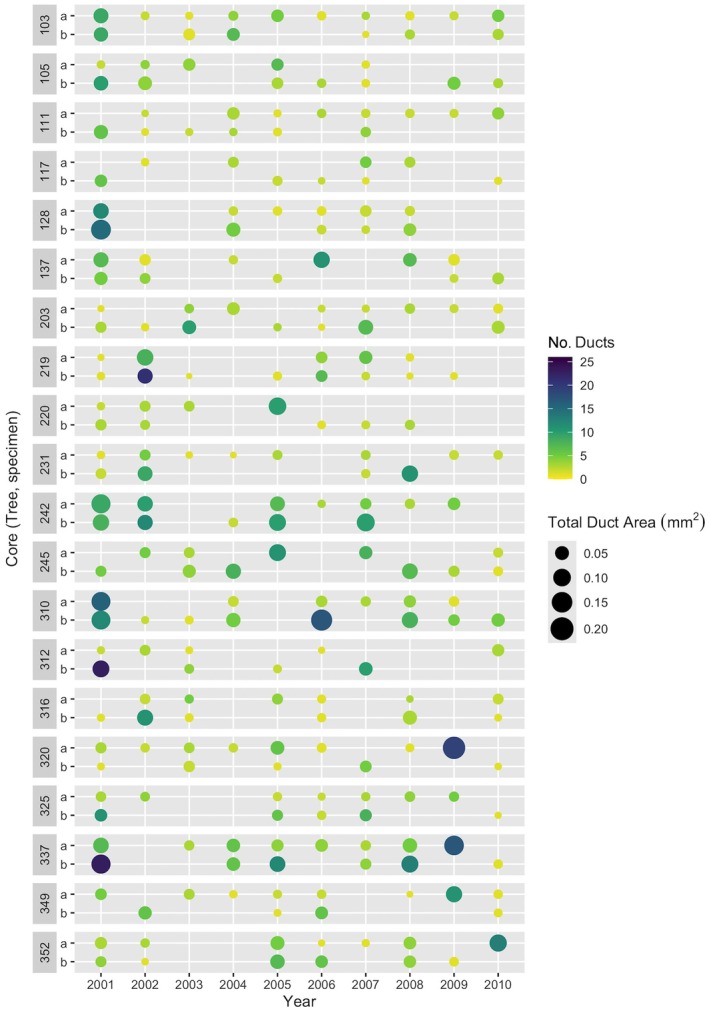
Dot plot comparing the resin duct production and total duct area from 2001 to 2010 measured within annual rings of paired cores (i.e., from the same tree). Two cores were removed from observed tamarack (*Larix laricina*) located in Beltrami Island State Forest, Minnesota during an outbreak of eastern larch beetle (*Dendroctonus simplex*). For visual clarity, only cores from 20 randomly selected study trees are included in this figure. If no ducts appear in a given year, no dot is drawn. Ten or more ducts occurring within a single annual ring (teal or blue dots) are likely traumatic in origin (Franceschi et al., 2005).

**FIGURE 3:**
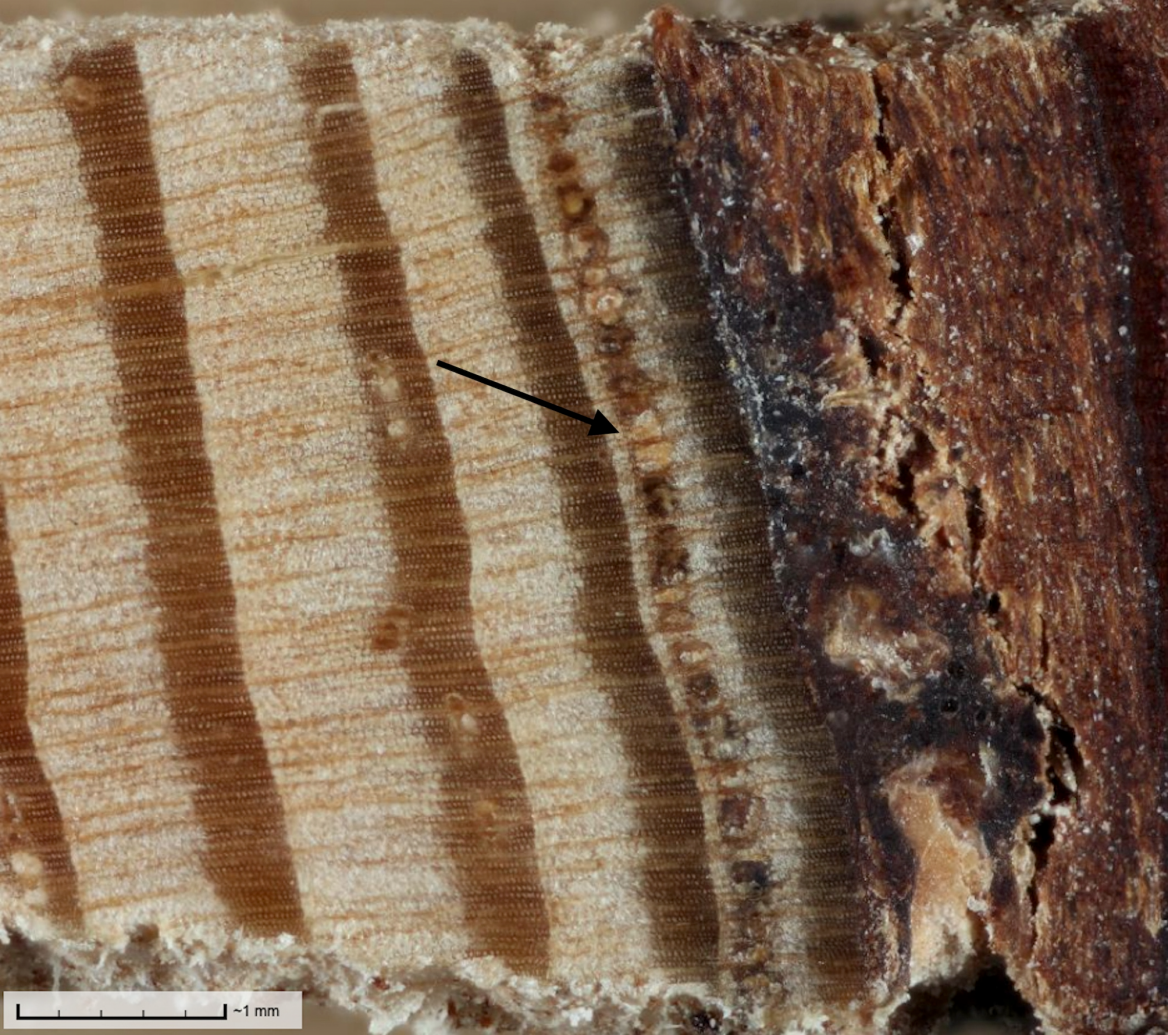
Tree core from a tamarack (*Larix laricina*) with a continuous line of resin ducts (black arrow) in the final annual ring corresponding to the year of colonization by eastern larch beetle (*Dendroctonus simplex*) during an outbreak in northern Minnesota. Such ducts are likely traumatic in origin (Franceschi et al., 2005). Image is located on the DendroElevator (http://dendro.elevator.umn.edu) platform. Photo credit: Grace Graham.

With tree cores, cross‐dating was deemed feasible and tree growth patterns were found to be similar within sites, with series intercorrelations close to 0.7. Locally absent rings were not detected using methods of cross‐dating. Resin ducts were generally small and infrequent in cores (Figure 1a, Table 1). On average, resin ducts were smaller than 0.008 mm^2^ and accounted for less than 0.5% of the area of annual rings. Although mean duct production seemed consistent across trees with between 4 and 6 ducts per annual ring, pooling duct data across 2 cores and 10 annual rings for each tree dampened the extreme variability in duct numbers (Figure 2). Core wood without any ducts was common: 37.8% of annual rings from 2001 to 2011 did not have a single resin duct. All cumulative resin duct metrics were skewed by annual rings that contained large clusters of tangentially aligned resin ducts (Figure 3) that are likely traumatic in origin (Franceschi et al., 2005). These features could contain as many as 40 individual resin ducts and contribute up to 16.3% of the area of an annual ring. These older injury responses were occasionally highly localized with numerous traumatic ducts appearing in only one of the two cores taken from the same tree (Figure 2).

### Relationships between resin traits and tree characteristics

A full analysis of relationships between xylem resin ducts in tree cores, phloem traits, and overall characteristics of trees is included in Appendix S3. In brief, BAI and ring width were both good predictors for absolute resin capacity—those trees with faster recent growth rates also had the largest ducts, greatest total duct area, and most numerous ducts (Appendix S3: Table S1). Phloem thickness had a number of significant relationships with various tree and resin duct traits, but most of these trends disappeared with the inclusion of dbh in the model (Appendix S3: Table S2). The best model for predicting phloem thickness included terms for both size (dbh) and growth rate (BAI): Those trees with a larger dbh and higher recent growth rates had the thickest phloem. However, smaller trees had higher densities of preformed phloem resin cells (Appendix S3: Table S3). This defensive metric was not related to any of the xylem resin duct traits measured in tree cores (Appendix S3: Table S3).

### Patterns of host selection by outbreaking bark beetles

**FIGURE 4:**
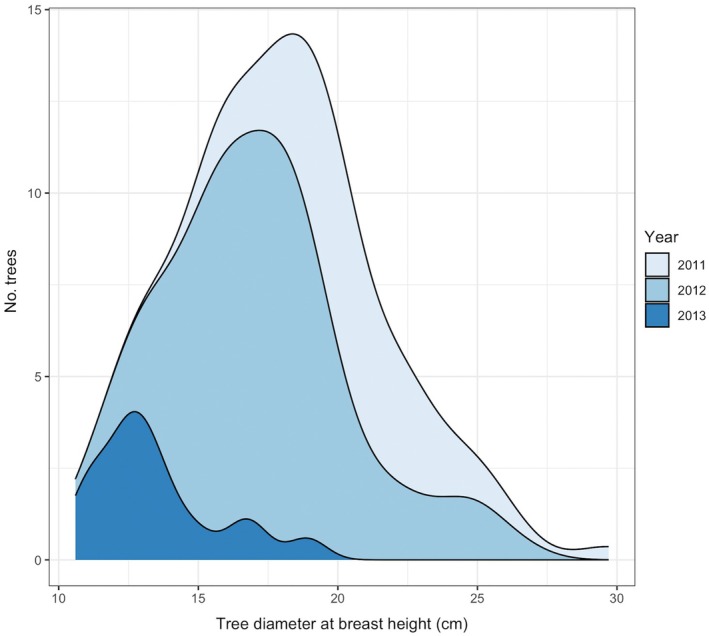
A smoothed histogram representing the size distribution of living study trees present in sites at the beginning of each indicated year before eastern larch beetle (*Dendroctonus simplex*) moved through the stands and killed trees. All trees are tamarack (*Larix laricina*) found in Beltrami Island State Forest, Minnesota.

As the beetles culled trees from the forest, they successively removed the largest trees (Figure 4). In 2011 and 2012, during the early stages of the outbreak, tree diameter was the best predictor of whether a tree would be colonized: Trees of larger diameters had a greater likelihood of colonization (Tables 3 and 4). Upon initial entry in the stand in 2011, beetles attacked trees with faster recent growth rates (i.e., higher 5‐ and 10‐year BAI), but these metrics were highly correlated with overall tree diameter (Appendix S3: Table S4) and did not contribute additional explanatory power when combined with dbh in models. There were several additional traits exhibiting significant relationships to colonization patterns in 2012, but none of these variables retained predictive power when dbh was added as a fixed effect (Table 4). By 2013, when beetles had culled the majority of trees, no measured trait aligned with colonization patterns among the remaining trees (Table 5). A small diameter tree heavily attacked in late summer 2012 was removed as an outlier from analysis, but speaks to the limited availability of host material at that time. Throughout these three outbreak years, attack densities of ELB were highest on trees with thicker phloem and faster recent growth rates (Table 6). All other significant variables, including dbh, lost explanatory power when either BAI or phloem thickness was included as fixed effects.

**TABLE 3:** Relationships between tamarack (*Larix laricina*) characteristics and the likelihood of successful colonization by eastern larch beetle (*Dendroctonus simplex*) within the first summer (2011) of beetle entry into observed stands in northern Minnesota.

Explanatory variable	Intercept				Slope				ΔAIC
	Estimate	SE	*Z*	*p*	Estimate	SE	*Z*	*p*	
Intercept only	−1.01	0.2	−5.11	<0.0001	…	…	…	…	29.3
**dbh**	−7.75	1.5	−5.15	<0.0001	0.36	0.08	4.66	<0.0001	0
Age	−2.32	1.34	−1.74	0.0828	0.03	0.03	1	0.3178	30.3
Stand density	−0.66	0.46	−1.43	0.154	−0.02	0.02	−0.84	0.403	30.6
Ring width 5	−1.08	0.39	−2.76	0.0057	0.04	0.18	0.2	0.8401	31.2
Ring width 10	−1.25	0.45	−2.78	0.0055	0.14	0.23	0.6	0.5501	30.9
**BAI 5** ^a^	−1.62	0.36	−4.43	<0.0001	5.06E‐04	2.45E‐04	2.07	0.0384	27
**BAI 10** ^a^	−2.06	0.49	−4.19	<0.0001	9.67E‐04	4.04E‐04	2.39	0.0168	22.9
Duct production 5	−0.82	0.45	−1.82	0.0695	−0.04	0.09	−0.45	0.6496	31.1
Duct production 10	−1.79	0.53	−3.36	0.0008	0.15	0.09	1.63	0.1042	28.6
Duct size 5	−1.68	0.85	−1.96	0.0499	95.88	118.34	0.81	0.4178	30.5
Duct size 10	−2.4	0.9	−2.66	0.0079	205.8	128.44	1.6	0.1091	28.8
Total duct area 5	−0.99	0.39	−2.55	0.0108	−0.49	10.27	−0.05	0.9618	31.3
Total duct area 10	−1.76	0.46	−3.85	0.0012	22.24	11.75	1.89	0.0584	27.7
Duct density 5	−0.81	0.31	−2.62	0.0088	−0.4	0.49	−0.81	0.4176	30.6
Duct density 10	−0.99	0.32	−3.11	0.0019	−0.03	0.49	−0.67	0.9469	31.3
Relative duct area 5	−1	0.33	−3.07	0.0021	−0.01	0.4	−0.03	0.9746	31.3
Relative duct area 10	−1.35	0.36	−3.79	0.0002	0.51	0.43	1.19	0.2361	29.9
Phloem thickness	−5.02	1.77	−2.83	0.0046	0.8	0.54	1.47	0.141	.
Phloem resin cell density	−2.26	0.79	−2.87	0.0042	−0.06	0.12	−0.48	0.6307	.

*Note*: All 131 trees characterized here were located in an epicenter of beetle activity in Beltrami Island State Forest, Minnesota. Estimates are based on generalized linear mixed‐effects models. In this year, phloem samples (*n* = 102) were not obtained for all trees so phloem thickness and phloem resin cell density do not have comparable Akaike information criterion (AIC) values. Explanatory variables in bold typeface have slope *p*‐values below a 0.05 threshold for statistical significance. A full description of variables can be found in Table 2.

Abbreviations: BAI, basal area increment; dbh, diameter at breast height.

^a^ Data are log‐transformed.

**TABLE 4:** Relationships between tamarack (*Larix laricina*) characteristics and the likelihood of successful colonization by eastern larch beetle (*Dendroctonus simplex*) within the second summer (2012) of beetle activity in observed stands in northern Minnesota.

Explanatory variable	Intercept				Slope				ΔAIC
	Estimate	SE	*Z*	*p*	Estimate	SE	*Z*	*p*	
Intercept only	2.05	0.73	2.8	0.0052	…	…	…	…	22.1
**dbh**	−7.68	2.61	−2.94	0.0033	0.62	0.17	3.61	0.0003	0
**Age**	−5.41	2.66	−2.05	0.0403	0.18	0.07	2.72	0.0065	16.6
Stand density	1.33	1.1	1.22	0.224	0.04	0.05	0.78	0.438	23.5
Ring width 5	2.16	0.87	2.47	0.0135	−0.06	0.25	−0.22	0.8253	24.1
Ring width 10	2.08	0.92	2.27	0.0232	−0.02	0.35	−0.06	0.9562	24.1
BAI 5^a^	1.4	0.88	1.59	0.113	6.55E‐04	4.74E‐04	1.38	0.167	21.7
BAI 10^a^	1.05	0.93	1.13	0.2603	1.24E‐03	7.07E‐04	1.75	0.0797	20
Duct production 5	1.24	0.95	1.31	0.19	0.15	0.12	1.31	0.191	22.3
Duct production 10	0.83	1.03	0.8	0.422	0.26	0.16	1.61	0.108	21.3
**Duct size 5**	−1.28	1.44	−0.89	0.3754	511.57	200.48	2.55	0.0107	16.4
**Duct size 10**	−1.67	1.6	−1.04	0.298	611.14	244.39	2.5	0.0124	16
**Total duct area 5**	0.87	0.91	0.96	0.3377	33.72	16.71	2.02	0.0436	18.8
**Total duct area 10**	0.29	0.98	0.29	0.7701	61.31	25.04	2.45	0.0143	16.4
Duct density 5	2.03	0.83	2.45	0.0144	0.03	0.63	0.05	0.9567	24.1
Duct density 10	2.18	0.92	2.36	0.0182	−0.22	0.91	−0.25	0.8043	24.1
Relative duct area 5	1.12	0.86	1.3	0.193	1.43	0.92	1.56	0.12	20.6
Relative duct area 10	1.02	0.93	1.1	0.273	1.6	1.13	1.42	0.157	21.6
Phloem thickness	0.08	1.43	0.06	0.955	0.74	0.45	1.63	0.103	21.2
**Phloem resin cell density**	3.04	0.89	3.43	0.0006	−0.15	0.08	−1.98	0.0472	19.8

*Note*: The 96 trees characterized here were located in an epicenter of beetle activity in Beltrami Island State Forest, Minnesota. Estimates are based on generalized linear mixed‐effects models. Explanatory variables in bold typeface have slope *p*‐values below a 0.05 threshold for statistical significance. A full description of variables can be found in Table 2.

Abbreviations: AIC, Akaike information criterion; BAI, basal area increment; dbh, diameter at breast height.

^a^ Data are log‐transformed.

**TABLE 5:** Relationships between tamarack (*Larix laricina*) characteristics and the likelihood of successful colonization by eastern larch beetle (*Dendroctonus simplex*) within the third summer (2013) of beetle activity in observed stands in northern Minnesota.

Explanatory variable	Intercept				Slope				ΔAIC
	Estimate	SE	*Z*	*p*	Estimate	SE	*Z*	*p*	
Intercept only	−0.61	0.51	−1.19	0.232	…	…	…	…	4.3
dbh	−1.97	3.14	−0.63	0.531	0.1	0.23	0.44	0.659	6.1
Age	−47.77	24.96	−1.91	0.0557	0.73	0.42	1.73	0.0835	0
Stand density	1.79	1.49	1.2	0.231	−0.19	0.12	−1.64	0.101	3
Ring width 5	−0.97	5.51	−0.18	0.8608	−1.84	1.1	−1.67	0.0953	2.9
Ring width 10	−1.06	5.31	−0.2	0.842	−1.76	1.19	−1.49	0.138	4
BAI 5^a^	0.07	0.96	0.07	0.945	−7.53E‐04	0	−0.79	0.431	5.5
BAI 10^a^	−0.16	−0.16	0.95	0.87	−6.01E‐04	−0.55	0	0.586	6
Duct production 5	−0.73	0.97	−0.75	0.456	0.02	0.12	0.15	0.884	6.3
Duct production 10	−0.87	1.37	−0.64	0.524	0.05	0.23	0.21	0.832	6.2
Duct size 5	−0.44	2.06	−0.21	0.832	−25.97	305.41	−0.09	0.932	6.3
Duct size 10	−2.08	2.49	−0.84	0.404	239.04	390.61	0.61	0.541	5.9
Total duct area 5	−0.24	0.82	−0.3	0.767	−7.68	14.29	−0.54	0.591	6
Total duct area 10	−0.22	1.07	−0.21	0.835	−10.95	27.51	−0.4	0.691	6.1
Duct density 5	−0.77	0.81	−0.96	0.339	0.21	0.77	0.27	0.787	6.2
Duct density 10	−0.64	0.81	−0.79	0.428	0.05	0.95	0.05	0.957	6.3
Relative duct area 5	−0.77	0.77	−1	0.32	0.16	0.56	0.29	0.774	6.2
Relative duct area 10	−0.85	0.94	−0.91	0.363	0.31	0.98	0.32	0.751	6.2
Phloem thickness	−1.69	2.54	−0.66	0.506	0.4	0.92	0.44	0.662	6.1
Phloem resin cell density	0.4	1.17	0.34	0.732	−0.09	0.1	−0.92	0.358	5.4

*Note*: The 17 trees characterized here were located in an epicenter of eastern larch beetle (ELB) activity in Beltrami Island State Forest, Minnesota. Estimates are based on generalized linear mixed‐effects models. A full description of variables can be found in Table 2.

Abbreviations: AIC, Akaike information criterion; BAI, basal area increment; dbh, diameter at breast height.

^a^ Data are log‐transformed.

**TABLE 6:** Relationships between attack density (attack points/100 cm^2^) of eastern larch beetles (*Dendroctonus simplex*) and host tamarack (*Larix laricina*) characteristics in observed stands in northern Minnesota.

Explanatory variable	Intercept					Slope					ΔAIC
	Estimate	SE	*T*	df	*p*	Estimate	SE	*t*	df	*p*	
Intercept only	2.1	0.19	11.07	3	0.0025	…	…	…	…	…	20.1
**dbh**	0.68	0.3	2.31	45	0.0256	0.08	0.02	5.19	48	<0.0001	1.7
Age	2.8	0.77	3.65	11	0.0038	−0.02	0.02	−0.94	12	0.3649	21.6
**Stand density**	2.64	0.28	9.34	7	<0.0001	−0.04	0.01	−3.7	48	0.0006	10.8
**Ring width 5**	1.65	0.15	10.89	7	<0.0001	0.25	0.05	4.63	48	<0.0001	6.9
**Ring width 10**	1.49	0.15	9.66	13	<0.0001	0.38	0.08	4.92	47	<0.0001	4.9
**BAI 5** ^a^	−1.04	0.59	−1.76	51	0.0837	0.46	0.09	5.41	51	<0.0001	1.8
**BAI 10** ^a^	−1.46	0.64	−2.3	51	0.0258	0.53	0.09	5.67	51	<0.0001	0
Duct production 5	2.15	0.26	8.25	8	<0.0001	−0.01	0.03	−0.25	50	0.803	20.9
Duct production 10	1.86	0.27	6.81	12	<0.0001	0.05	0.04	1.28	48	0.206	19.4
**Duct size 5**	1.49	0.33	4.55	20	0.0002	87.29	36.1	2.42	46	0.0196	15.4
**Duct size 10**	1.38	0.35	3.91	28	0.0005	111.16	45.18	2.46	46	0.0177	15.2
Total duct area 5	2.12	0.24	8.91	6	0.0002	−0.23	2.9	−0.08	49	0.9383	20.9
Total duct area 10	1.93	0.24	7.93	8	<0.0001	5.43	4.35	1.25	48	0.218	19.4
**Duct density 5**	2.31	0.18	12.59	3	0.0006	−0.28	0.12	−2.26	48	0.0287	16.3
Duct density 10	2.34	0.21	4.88	11	0.0001	−0.38	0.19	−1.93	48	0.0597	17.6
Relative duct area 5	2.26	0.19	11.77	3	0.0006	−0.15	0.09	−1.79	47	0.0794	18
Relative duct area 10	2.27	0.22	10.33	5	0.0001	−0.2	0.15	−1.36	47	0.18	19.2
**Phloem thickness**	0.64	0.26	2.46	29	0.0201	0.46	0.08	5.52	37	<0.0001	…
Phloem resin cell density	2.09	0.24	8.84	7	<0.0001	−0.01	0.02	−0.6	38	0.552	…

*Note*: All 51 trees characterized here were located in Beltrami Island State Forest, Minnesota, and attacked by beetles between the summers of 2011 and 2013. Estimates are based on linear mixed‐effects models. Phloem samples (*n* = 41) were not obtained for all trees so phloem thickness and phloem resin cell density do not have comparable Akaike information criterion (AIC) values. Explanatory variables in bold typeface have slope *p*‐values below a 0.05 threshold for statistical significance. A full description of variables can be found in Table 2.

Abbreviations: BAI, basal area increment; dbh, diameter at breast height.

^a^ Data are log‐transformed.

### Reproductive rate of beetles following successful colonization of trees

Tree traits that predicted colonization success such as phloem thickness or BAI did not ultimately predict brood emergence per 100‐cm^2^ bark surface area (Table 7) or brood production per parent female (Table 8). Instead, once the tree was colonized, phloem resin cell density emerged as the best predictor of brood success. A greater number of resin cells per unit area of phloem corresponded to a diminished brood adult density per tree and per parent female. Although dbh and tree age each appeared to play a role in brood density or female reproductive rate respectively, these trends disappeared with the addition of phloem resin cell density in models.

**TABLE 7:** Relationships between brood emergence (brood/100 cm^2^ ^a^) of eastern larch beetles (*Dendroctonus simplex*) and host tamarack (*Larix laricina*) characteristics in observed stands in northern Minnesota.

Explanatory variable	Intercept					Slope					ΔAIC
	Estimate	SE	*T*	df	*p*	Estimate	SE	*t*	df	*p*	
Intercept only	2.94	0.27	10.83	2	0.0093	…	…	…	…	…	5.6
**dbh**	1.32	0.55	2.41	52	0.0194	0.09	0.03	3.14	52	0.0028	0
Age	1.68	1.09	1.55	51	0.128	0.03	0.03	1.21	52	0.234	6.4
Stand density	2.69	0.35	7.77	7	<0.0001	0.02	0.02	1.07	50	0.289	6.5
Ring width 5	2.85	0.33	8.67	5	0.0005	0.05	0.11	0.44	48	0.6626	7.4
Ring width 10	2.81	0.36	7.77	7	0.0001	0.08	0.15	0.51	48	0.6138	7.4
BAI 5^b^	1.63	1.2	1.35	52	0.182	0.19	0.17	1.12	51	0.266	6.5
BAI 10^b^	0.95	1.33	0.72	27	0.478	0.3	0.19	1.55	36	0.13	6
Duct production 5	2.65	0.42	6.37	7	0.0004	0.04	0.05	0.98	51	0.3308	6.7
Duct production 10	2.51	0.44	5.74	12	<0.0001	0.08	0.06	1.25	48	0.219	6.1
Duct size 5	2.32	0.53	4.38	27	0.0002	87.3	65.8	1.33	49	0.1908	5.9
Duct size 10	2.01	0.6	3.36	34	0.0019	141.45	81.6	1.73	48	0.0895	4.7
Total duct area 5	2.52	0.37	6.87	5	0.0011	9.33	4.72	1.98	50	0.0535	3.9
Total duct area 10	2.33	0.36	6.42	8	0.0002	16.95	7.07	2.4	48	0.0204	2.2
Duct density 5	2.88	0.32	9	3	0.0018	0.07	0.22	0.34	49	0.7384	7.5
Duct density 10	2.84	0.35	8.19	5	0.0006	0.16	0.34	0.47	48	0.6426	7.4
Relative duct area 5	2.81	0.32	8.75	3	0.0023	0.13	0.15	0.85	49	0.3994	6.9
Relative duct area 10	2.66	0.35	7.68	5	0.0008	0.34	0.25	1.36	48	0.1815	5.8
Phloem thickness	2.16	0.58	3.74	42	0.0006	0.25	0.18	1.38	42	0.1748	…
**Phloem resin cell density**	3.62	0.29	12.45	42	<0.0001	−0.1	0.04	−2.76	42	0.0085	…

*Note*: All 52 trees characterized here were located in Beltrami Island State Forest, Minnesota, and attacked by beetles between the summers of 2011 and 2013. Estimates are based on linear mixed‐effects models. Phloem samples (*n* = 42) were not obtained for all trees so phloem thickness and phloem resin cell density do not have comparable Akaike information criterion (AIC) values. Explanatory variables in bold typeface have slope *p*‐values below a 0.05 threshold for statistical significance. A full description of variables can be found in Table 2.

Abbreviations: BAI, basal area increment; dbh, diameter at breast height.

^a^ Data are square‐root‐transformed.

^b^ Data are log‐transformed.

**TABLE 8:** Relationships between reproductive success (brood/adult female^a^) of eastern larch beetles (*Dendroctonus simplex*) and host tamarack (*Larix laricina*) characteristics in observed stands in northern Minnesota.

Explanatory variable	Intercept					Slope					ΔAIC
	Estimate	SE	*T*	df	*p*	Estimate	SE	*t*	df	*p*	
Intercept only	1.72	0.08	20.86	52	<0.0001	…	…	…	…	…	2.3
dbh	1.22	0.35	3.5	52	0.001	0.03	0.02	1.46	52	0.1512	2.2
**Age**	0.4	0.63	0.63	52	0.5318	0.03	0.01	2.11	52	0.0401	0
Stand density	1.42	0.17	8.44	52	<0.0001	0.02	0.01	1.99	52	0.0523	0.5
Ring width 5	1.82	0.15	12.37	52	<0.0001	−0.06	0.07	−0.85	52	0.401	3.6
Ring width 10	1.83	0.18	10.43	52	<0.0001	−0.07	0.09	−0.74	52	0.465	3.7
BAI 5^b^	1.79	0.73	2.46	52	0.0174	−0.01	0.11	−0.1	52	0.9209	4.3
BAI 10^b^	1.61	0.8	2.02	52	0.0487	0.02	0.12	0.13	52	0.8955	4.3
Duct production 5	1.66	0.19	8.69	52	<0.0001	0.01	0.03	0.31	52	0.759	4.2
Duct production 10	1.62	0.23	7.1	52	<0.0001	0.02	0.04	0.44	52	0.662	4.1
Duct size 5	1.63	0.31	5.31	52	<0.0001	11.67	41.52	0.28	52	0.78	4.2
Duct size 10	1.55	0.35	4.38	52	<0.0001	25.18	52.25	0.48	52	0.632	4.1
Total duct area 5	1.56	0.16	10.02	52	<0.0001	3.52	2.93	1.2	52	0.235	2.9
Total duct area 10	1.48	0.18	8	52	<0.0001	6.59	4.56	1.44	52	0.155	2.2
Duct density 5	1.62	0.13	12.8	52	<0.0001	0.14	0.14	1.01	52	0.316	3.3
Duct density 10	1.57	0.15	10.4	52	<0.0001	0.24	0.21	1.16	52	0.251	3
Relative duct area 5	1.62	0.12	13.34	52	<0.0001	0.1	0.09	1.08	52	0.285	3.1
Relative duct area 10	1.52	0.15	10.27	52	<0.0001	0.25	0.16	1.58	52	0.12	1.8
Phloem thickness	1.79	0.34	5.24	42	<0.0001	−0.03	0.11	−0.25	42	0.802	…
**Phloem resin cell density**	2.15	0.17	12.93	42	<0.0001	−0.06	0.02	−3.05	42	0.0039	…

*Note*: All 52 trees characterized here were located in Beltrami Island State Forest, Minnesota, and attacked by beetles between the summers of 2011 and 2013. Estimates are based on linear mixed‐effects models. Phloem samples (*n* = 42) were not obtained for all trees so phloem thickness and phloem resin cell density do not have comparable Akaike information criterion (AIC) values. Explanatory variables in bold typeface have slope *p*‐values below a 0.05 threshold for statistical significance. A full description of variables can be found in Table 2.

Abbreviations: BAI, basal area increment; dbh, diameter at breast height.

^a^ Data are square root transformed.

^b^ Data are log‐transformed.

## DISCUSSION

In tamarack, preformed resin structures in the xylem and phloem do not appear to influence tree colonization patterns of ELB at epidemic population levels. Although tamarack are among those conifers of the *Pinaceae* family that exhibit constitutive resin ducts (Bannan, 1936; Krokene, 2015), these preformed structures may not play as vital a role in defensive capacity for this species. Compared to pines, the resin systems of tamarack visible in tree cores featured constitutive ducts that were smaller (0.008 mm^2^ vs. 0.019–0.023 mm^2^ in ponderosa pine (*Pinus ponderosa*), Hood et al., 2015) and more irregularly distributed (Figure 1), with a greater contribution to duct production and total duct area from traumatic resin ducts (Figure 2; Bannan, 1936; Trapp & Croteau, 2001). One tree removed as an outlier with highly skewed duct production likely attributable to a traumatic response exemplifies this trend. Many cores contained tangential rows of resin ducts in the final year of growth that were left out of the analysis because of difficulties in consistent measurement of those often incomplete rings across specimens (Figure 3). These ducts would have contributed to the actual resin exudation experienced by colonizing beetles, and appear to be important in other non‐pine systems (DeRose et al., 2017). However, it was not possible to capture the timing or importance of an induced defense with our study design utilizing archived cores originally collected for the sole purpose of establishing tree age. Overall, it may not be appropriate to apply pooled constitutive resin metrics developed for studies of *Pinus* species to assessments of defensive capacity outside that genus, and the consolidation of such methodologies across conifers may prove challenging.

More generally, the use of axial duct characteristics as a surrogate for resin production should be approached with caution. In tamarack, extreme variability in duct number between paired tree cores and across time (Figure 2) exemplifies how narrow a window into whole stem structure these 5‐mm diameter tree core samples represent, especially when examining features that may be localized around injury sites and thus have an irregular vertical and horizontal distribution around the bole (Bannan, 1936; Bollschweiler et al., 2008). The apparent disconnect between preformed xylem resin systems visible in tree cores and preformed resin cells visible in phloem samples raises further uncertainty in the extrapolation of resin duct features measured in cores to represent tree defensive capacity. Future research should strive to understand the connections among visible duct structures in the xylem and phloem and realized preformed or induced resin production in tamarack and other conifers.

Researchers have noted the importance of evolutionary context for observed patterns of host defense and bark beetle behavior. Some have proposed that members of the genus *Pinus* have more highly developed constitutive defenses due to their longer history of contending with aggressive bark beetle behavior or multiple generations of beetles each year (Krokene, 2015; Labandeira et al., 2001; Lieutier, 2002). Other conifer groups that did not have the intense selective pressure imposed by evolutionarily and seasonally consistent attacks of bark beetles rely more on inducible defenses, which require less long‐term investment of resources and are still effective against endemic level threats (Krokene, 2015; Lieutier, 2002).

Tamarack trees often grow in nutrient poor environments and, in the Lake State Region, the species has a long history of local ELB populations persisting at endemic levels (Johnston, 1990; Langor & Raske, 1989). Reliance on inducible defenses may thus be advantageous. The positive relationships of resin duct size and number to dbh and recent BAI support the idea that tamarack do not invest in constitutive defenses at the expense of growth, even when ELB is at outbreak levels. The true cost of induced or constitutive defenses and its impact on tree growth or survival during bark beetle outbreaks is difficult to measure (Gershenzon, 1994; Lombardero et al., 2000; Stamp, 2003). Such relationships are further complicated by the resource availability and injury experiences of individual trees (Gaylord et al., 2007; Greenslit et al., 2024; Kichas et al., 2020; Soderberg et al., 2022) as exemplified by an outlier tree removed from analysis due to extremely low BAI. Further study is needed to confirm whether patterns of growth and defensive investment revealed here are consistent in tamarack trees across the different site conditions and disturbance histories of this geographically widespread tree species.

Regardless of tree defensive strategy, beetles from this population of ELB behaved in a manner consistent with pine‐associated *Dendroctonus* species deemed “aggressive,” targeting and successfully overwhelming the largest trees with the thickest phloem first (Boone et al., 2011; Cole & Amman, 1969; Sullivan, 2011). The latent potential for seeking and killing the largest and most vigorous trees is not often expressed in ELB (Langor & Raske, 1989; Wood, 1982) or other bark beetle species that target conifers known to rely on induced defenses (Raffa & Berryman, 1987). Our work indicates that such a trait was present in local endemic populations and may have been activated by a sufficient increase in beetle numbers during favorable climatic conditions (McKee, 2015). Such a phenomenon has been documented in other, better known, tree‐killing species (Kausrud et al., 2011; Lindgren & Raffa, 2013; Raffa et al., 2005; Wallin & Raffa, 2004). Although tamarack phloem resin slightly diminished reproductive success of ELB in this study, the extent of the impact of increased defenses in colonized trees on insect populations is unknown, especially as parent beetles can increase the total available reproductive material by targeting larger trees. Future tree defense studies should go beyond initial host choice and examine the multigenerational effects of beetles reproducing in more resinous substrates.

This ELB outbreak also illustrates the management challenges created by a history of reactive research shaped by human values. The most well‐developed bark beetle management recommendations in North America were developed from research on those *Dendroctonus* species that target pines (Fettig et al., 2007, 2022; Windmuller‐Campione et al., 2021). The colonization pattern of heightened populations of ELB and its effect on tamarack stand dynamics is evident in tree diameter distribution over the course of the outbreak (Figure 4). In some ways this established preference of ELB for large trees clarifies management decisions as dbh is easy to measure and host choice similarities among *Dendroctonus* species allow for generalization of traditional stand hazard assessments built on these other bark beetle systems (Crocker et al., 2016; Windmuller‐Campione et al., 2021). However, as with tree defenses, the ecological niches and management intensity of tamarack and pines are quite different (Burns & Honkala, 1990; Duncan, 1954; Minnesota Department of Natural Resources, 2013; Shore et al., 2006). Stand‐level approaches for building forest resilience are hampered in tamarack‐dominated ecosystems that are poorly understood, hydrologically complex, often uninhabitable for most other tree species, and limited in economic incentives (Duncan, 1954; Minnesota Department of Natural Resources, 2013; Shaunette, 2022).

The rapid speed of climate change reveals the many ways in which former management paradigms constrain current forest stewardship (Keenan, 2015). In the context of bark beetles, these include a legacy of vulnerable landscapes, a failure to address complex disturbance interactions at multiple scales, and an emphasis on those species and populations that conflict with human goals regularly enough to warrant the designation of “pest” (Logan, Régnière, and Powell 2003; Raffa et al., 2009; Keenan, 2015; Aukema et al., 2016; Windmuller‐Campione et al., 2021). Recent destructive insect invasions have spurred the formation of groups dedicated to predicting the next exotic agent that will imperil North American forests (Uden et al., 2023) and inspired institutional investments in collaborative planning with community incentives to detect and rapidly respond to such threats (Animal & Plant Health Inspection Service, 2024; Bliss‐Ketchum et al., 2021). Practitioners and researchers will need to be similarly proactive in identifying and preparing for those native species with the potential to devastate forests under climate warming.

## AUTHOR CONTRIBUTIONS

Original field work was completed by Fraser McKee. Material preparation and data collection were performed initially by Fraser McKee with subsequent steps undertaken by Grace Graham with the support of Daniel Griffin. Grace Graham took the lead in data analysis and manuscript writing with supervision by Brian Aukema and Marcella Windmuller‐Campione. All authors provided critical feedback and helped shape the concepts, research, analysis, and manuscript.

## CONFLICT OF INTEREST STATEMENT

The authors declare no conflicts of interest.

## Supporting information


Appendix S1.

Appendix S2.

Appendix S3.

## Data Availability

Data (Graham et al., 2025) are available in the Digital Repository of the University of Minnesota (DRUM) at https://doi.org/10.13020/9gn2-9y11.
