# Supplementary material for: Tree defenses, host choice, and reproductive success of a native bark beetle under novel outbreak conditions
Source: Ecol Appl. 2026 Jan 14;36(1):e70176. doi: 10.1002/eap.70176 (PMC12800731; doi:10.1002/eap.70176)

Ecological Applications

Tree defenses, host choice, and reproductive success of a native bark beetle under novel outbreak conditions

Grace Graham, Marcella Windmuller-Campione, Daniel Griffin, Fraser McKee, and Brian Aukema

Appendix S1: Additional Field Methods

Tree characteristics

Diameter at breast height was measured to the nearest 0.1 cm at 1.4 m for each study tree following selection in June 2011. Additionally, at the beginning of each year of monitoring, a 5 x 2 cm (H x W) rectangular phloem sample was removed at 1.4 meter above the ground from all non-attacked trees. Samples in 2011 were taken from the east aspect of the bole with repeat samples when applicable taken 5 cm away from first samples (2012) or on the west aspect of the bole (2013). Phloem was not sampled from those trees colonized in 2011 prior to site selection as beetle activity and tree defensive response would impact phloem quality. Phloem was differentiated from bark by color and texture. Phloem thickness was recorded as the mean value of measurements occurring at 0.5, 2.5, and 4.5 cm along the 5 cm length of sample. A Leica MZ6 microscope with real-time camera and digital micrometer (Leica Microsystems, Wetzler, Germany) was used to measure thickness to the nearest 0.01 mm. The number of resin cells (Figure 1) within phloem samples were counted on the same longitudinal section surface used for thickness measurements. Resin cell density was calculated by dividing this count by the cross-sectional area (mean phloem thickness x 5 cm) of the sample.

Local stand density was estimated for study trees by calculating the basal area of surrounding trees via variable radius plot. Plots were established with each study tree serving as plot center and all trees with breast height (1.4 m) bole diameters filling a BAF 10 Jim-Gem Cruz-All gauge (Forestry Suppliers, Jackson, MS, U.S.A., Prod. # 59795) were tallied. The competition factor was calculated by multiplying this tally by 10 (the gauge basal area factor) and converting the resulting basal area measure to m^2/ha.

Beetles attack dynamics

Beetle attack dynamics and reproductive success were recorded from 52 tamarack trees killed by eastern larch beetle. Trees were monitored every seven days during the growing season in each of 2011, 2012, and 2013. In 2011, monitoring began immediately after site selection in June. In 2012 and 2013, monitoring began in late April at least 2 weeks prior to beetle emergence. For all years, monitoring concluded in October after at least 2 weeks after cessation of beetle emergence. Monitoring consisted of a visual inspection of the lower 2.5 m of each tagged tree bole for beetle boring and frass accumulation. Once trees were attacked, 20 x 20 cm (in 2011) or 40 x 25 cm (in 2012 and 2013) observation windows were installed on the south aspect of the bole centered at 1.6 meters above the ground. These “windows” consisted of four push pins bordered with string, and were checked during weekly site assessment determine the timing of attacks. Attack points were marked with pins to prevent double counting. Colonization was deemed complete when no new attacks occurred between two successive sample dates.

Upon completion of colonization, observation windows were removed and screen cages were installed. We placed two screen cages 16.5 x 30 cm (W x H) in size on each of the north and south bole aspect centered at 1.8 m above the ground. The number of attack points on the 495 cm^2 of bark covered by each of the cages was recorded prior to their placement. We checked collection cups fitted on the screen cages twice weekly throughout the summer monitoring and again in the spring of the year following tree colonization. Screen cages were removed in the fall after two weeks without captures and were reinstalled in the spring two weeks prior to beetle emergence.

Figure S1: An example of the 5 x 2 cm (H x W orientation when removed from tree) phloem samples removed from tamarack (*Larix laricina*) observed during an outbreak of eastern larch beetle (*Dendroctonus simplex*) in northern Minnesota. Examples of phloem resin cells on the measured surface are indicated by black arrows. Example of measured phloem thickness is indicated with a black line on the far left of sample. Photo credit: Grace Graham.

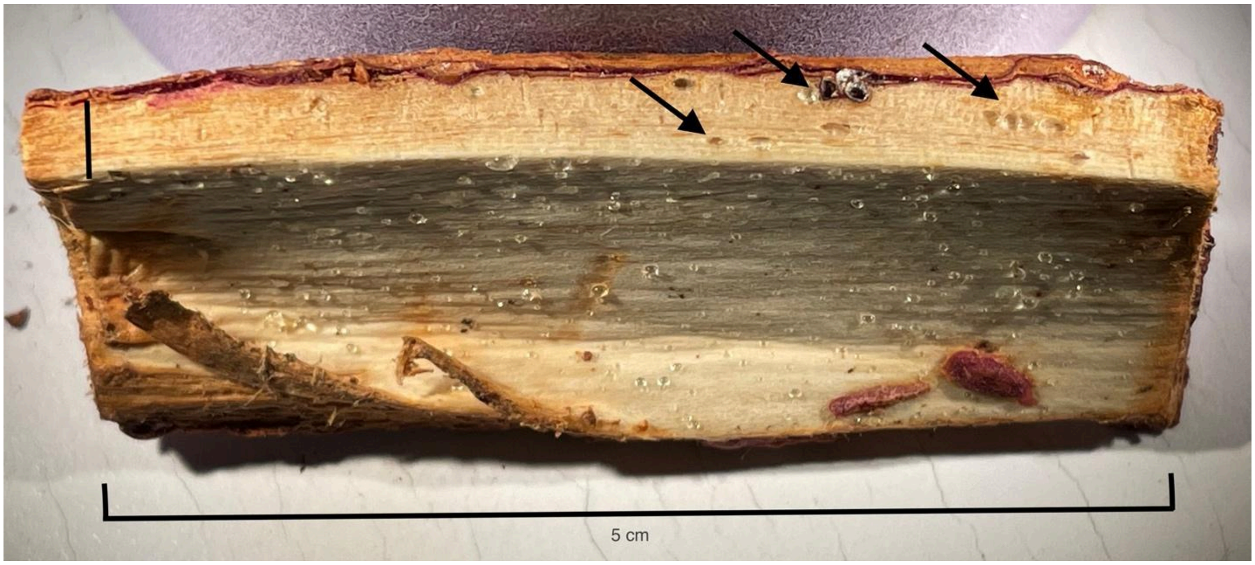

Supplement: Supplementary file 1 — Appendix S1. [file EAP-36-e70176-s003.pdf]
