# Supplementary material for: Tree defenses, host choice, and reproductive success of a native bark beetle under novel outbreak conditions
Source: Ecol Appl. 2026 Jan 14;36(1):e70176. doi: 10.1002/eap.70176 (PMC12800731; doi:10.1002/eap.70176)

Ecological Applications

Tree defenses, host choice, and reproductive success of a native bark beetle under novel outbreak conditions

Grace Graham, Marcella Windmuller-Campione, Daniel Griffin, Fraser McKee, and Brian Aukema

Appendix S2: Visual examples of dendrochronological methodologies

Figure S1 (next page): Five resin ducts embedded in the radial file of xylem tracheids visible in a high-resolution scan of a tamarack (*Larix laricina*) tree core collected during an outbreak of eastern larch beetle (*Dendroctonus simplex*) in northern Minnesota. (a) - General resin duct appearance with variation in color, size, and ring position. Note compression of surrounding tracheid cells. (b) - How these resin ducts would be measured using ellipse tools embedded in DendroElevator platform. Image is located on the DendroElevator (http://dendro.elevator.umn.edu) platform. Photo credit: Grace Graham.

Figure S1:

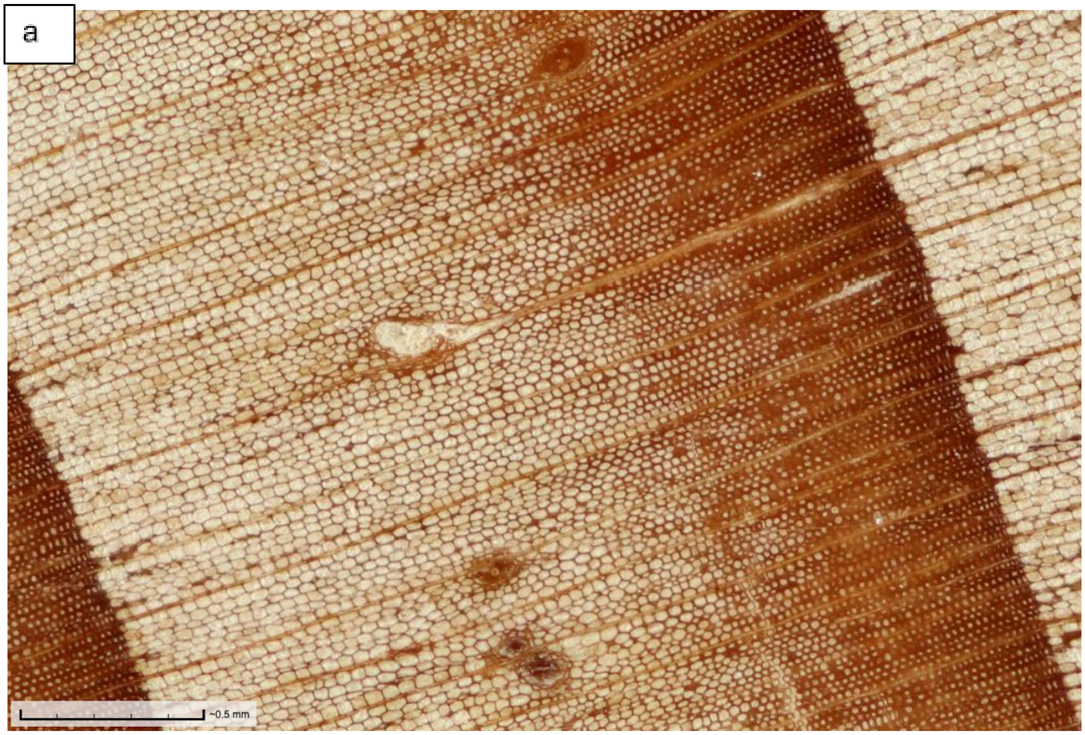

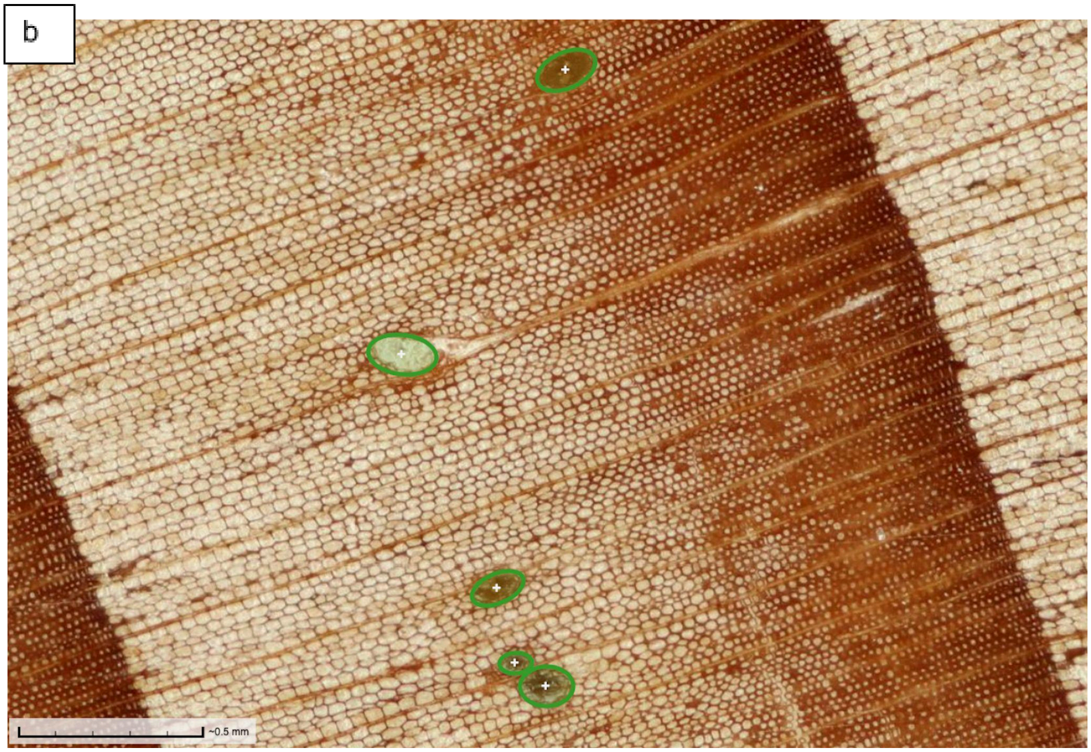

Figure S2: A representative image of a tree core with measurement area highlighted. Blue guidelines are 4 mm apart and begin in the annual ring corresponding to the year 2001. All ellipses with center points (white crosses) appearing within these guidelines were included in our analysis. The tree core is from a tamarack (*Larix laricina*) located in Beltrami Island State Forest, Minnesota observed during an outbreak of eastern larch beetle (*Dendroctonus simplex*). Image is located on the DendroElevator (http://dendro.elevator.umn.edu) platform. Photo credit: Grace Graham.

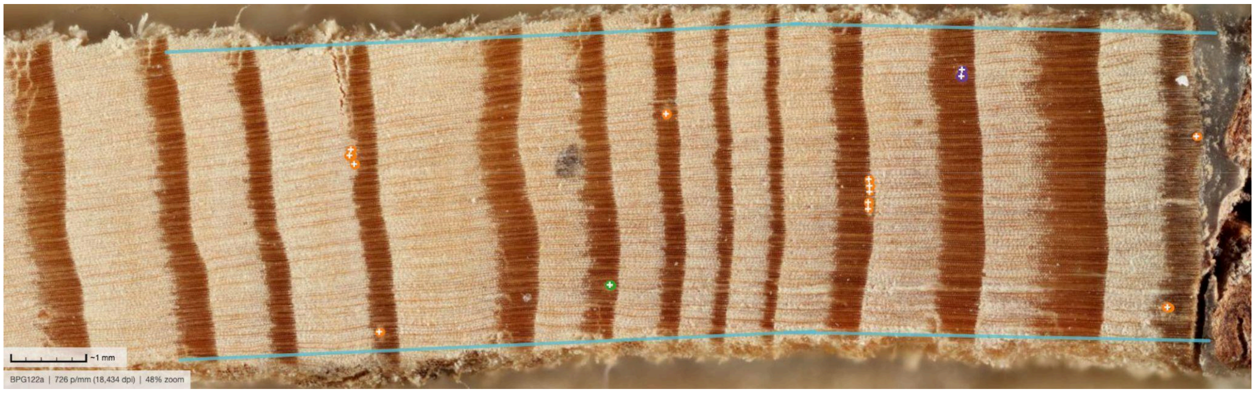

Figure S3: Information tagged to an ellipse drawn around a single resin duct in the DendroElevator platform. The top value corresponds to the year in which the resin duct occurs with a decimal to represent ring position (1: first line of tracheids; 2: earlywood; 3: transition between earlywood and latewood; 4: latewood). This duct is positioned in the latewood of 2008. The bottom value is the area of the ellipse in mm^2. The tree core is from a tamarack (*Larix laricina*) located in Beltrami Island State Forest, Minnesota observed during an oubreak of eastern larch beetle (*Dendroctonus simplex*). Image is located on the DendroElevator (http://dendro.elevator.umn.edu) platform. Photo credit: Grace Graham.

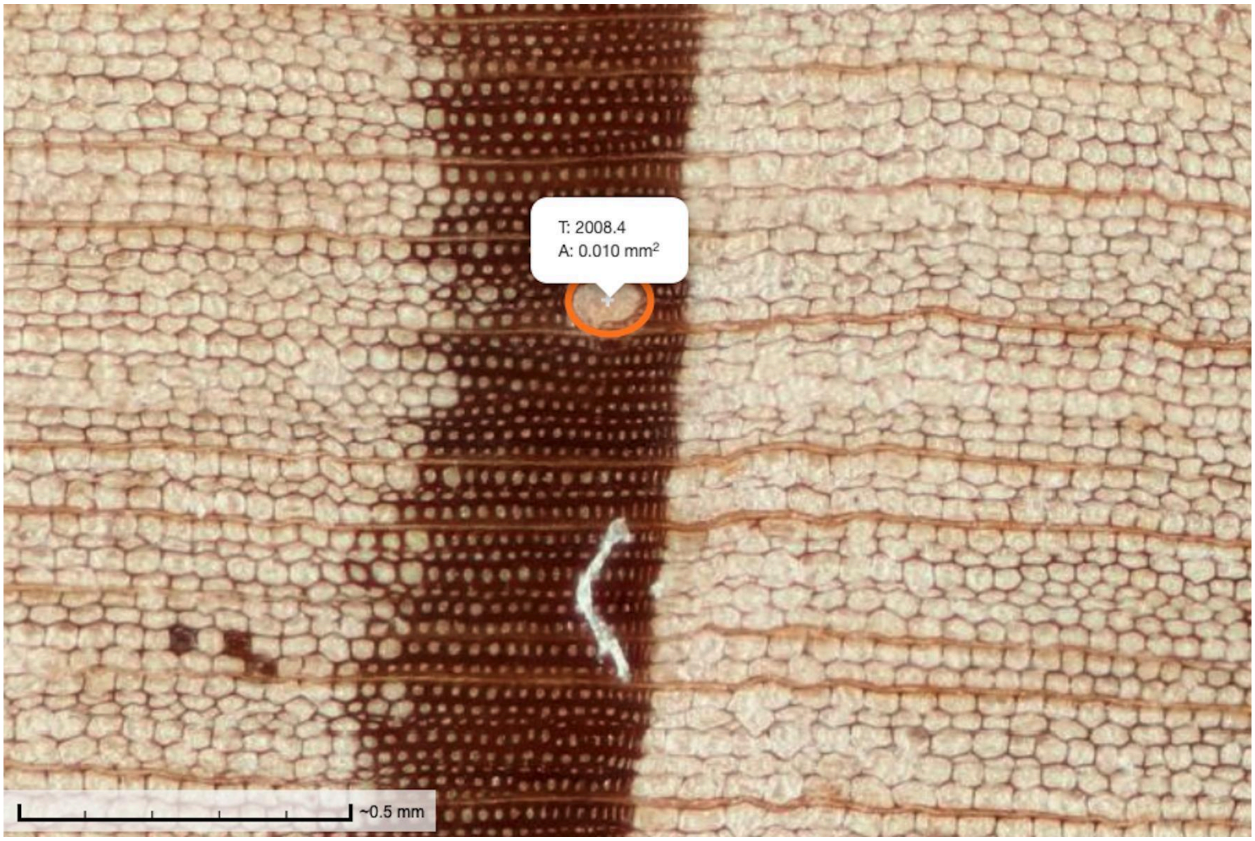

Supplement: Supplementary file 2 — Appendix S2. [file EAP-36-e70176-s001.pdf]
