# Supplementary material for: Tree defenses, host choice, and reproductive success of a native bark beetle under novel outbreak conditions
Source: Ecol Appl. 2026 Jan 14;36(1):e70176. doi: 10.1002/eap.70176 (PMC12800731; doi:10.1002/eap.70176)
Supplement: Supplementary file 3 — Appendix S3. [file EAP-36-e70176-s002.pdf]

Ecological Applications

Tree defenses, host choice, and reproductive success of a native bark beetle under novel outbreak conditions

Grace Graham, Marcella Windmuller-Campione, Daniel Griffin, Fraser McKee, and Brian Aukema

Appendix S3: Results regarding relationships among tree characteristics

Table S1: Relationships between unstandardized constitutive resin traits in tree cores and other tree characteristics in tamarack (*Larix laricina*) observed during an outbreak of eastern larch beetle (*Dendroctonus simplex*) based on linear mixed effects models.

Response Variable	Explanatory Variable	Intercept					Slope					
		EST	SE	*t*	*df*	*p*	EST	SE	*t*	*df*	*p*	ΔAIC
Duct size 10[a]	Intercept only	0.08	8.14 E-04	101.00	129.0	< 0.0001	.	.	.	.	.	30.1
	**DBH**	0.07	3.90 E-03	17.24	129.0	< 0.0001	8.38 E-04	2.13 E-04	3.93	129.0	0.0001	17.5
	Age	0.09	0.01	15.74	129.0	< 0.0001	-9.40 E-05	1.28 E-04	-0.73	129.0	0.4650	31.5
	Stand density	0.09	0.00	45.19	129.0	< 0.0001	-1.77 E-04	9.91 E-05	-1.78	129.0	0.0770	28.9
	**Ring width 10**	0.07	1.81E-03	41.23	15.3	< 0.0001	4.40 E-03	9.22 E-04	4.77	55.1	< 0.0001	11.9
	**BAI 10[b]**	0.03	0.01	3.24	102.0	0.0016	0.01	1.28 E-03	6.23	107.0	< 0.0001	0.0
Total duct area 10[a]	Intercept only	0.18	0.01	28.34	2.7	0.0002	.	.	.	.	.	14.1
	**DBH**	0.13	0.02	6.62	101.0	< 0.0001	2.79 E-03	1.07 E-03	2.60	127.0	0.0105	9.5
	Age	0.22	0.03	7.18	5.9	0.0004	-7.65 E-04	7.02 E-04	-1.09	6.7	0.3134	15.4
	**Stand density**	0.21	0.01	16.97	12.6	< 0.0001	-1.26 E-03	5.33 E-04	-2.36	111.0	0.0200	10.8
	**Ring width 10**	0.15	0.01	17.45	129.0	< 0.0001	0.02	4.57 E-03	3.95	129.0	0.0001	2.9
	**BAI 10[b]**	-0.01	0.04	-0.19	129.0	0.8510	0.03	0.01	4.36	129.0	< 0.0001	0.0
Duct production 10[a]	Intercept only	2.24	0.08	29.32	2.8	0.0001	.	.	.	.	.	2.4
	DBH	1.97	0.21	9.22	85.8	< 0.0001	0.02	0.01	1.35	126.4	0.1800	2.6
	Age	2.59	0.33	7.77	7.5	0.0001	-0.01	0.01	-1.06	8.6	0.3170	3.8
	**Stand density**	2.45	0.13	18.94	11.7	< 0.0001	-0.01	0.01	-2.13	117.2	0.0352	0.0
	**Ring width 10**	2.04	0.10	20.82	15.1	< 0.0001	0.12	0.05	2.38	59.7	0.0206	0.3
	**BAI 10[b]**	1.20	0.49	2.46	99.8	0.0158	0.15	0.07	2.14	105.5	0.0349	0.6

All 129 trees characterized here are tamarack (*Larix laricina*) located in stands impacted by ELB in Beltrami Island State Forest, Minnesota. [a] data are square root transformed; [b] data are

log transformed. Explanatory variables in bold typeface have slope p-values below a 0.05 threshold for statistical significance. DBH refers to diameter at breast height and BAI refers to basal area increment. A full description of variables can be found in Table 2 of the primary manuscript.

Table S2: Relationships between phloem thickness and other tree characteristics in tamarack (*Larix laricina*) observed during an outbreak of eastern larch beetle (*Dendroctonus simplex*) based on linear mixed effects models.

Response Variable	Explanatory Variable	Intercept					Slope					ΔAIC
		EST	SE	*t*	*df*	*p*	EST	SE	*t*	*df*	*p*	
Phloem thickness	.	2.91	0.25	11.59	3.4	0.0007	.	.	.	.	.	57.9
	**DBH**	0.67	0.29	2.34	37.9	0.0249	0.13	0.01	9.31	97.7	< 0.0001	0.0
	Age	3.75	0.67	5.62	18.7	< 0.0001	-0.02	0.02	-1.33	25.9	0.1960	58.8
	**Stand density**	3.69	0.29	12.83	7.3	< 0.0001	-0.05	0.01	-5.54	98.4	< 0.0001	33.3
	**Ring width 5**	2.32	0.20	11.54	3.6	0.0005	0.32	0.06	5.64	56.2	< 0.0001	36.2
	**Ring width 10**	2.14	0.20	10.52	4.3	0.0003	0.47	0.08	6.26	39.1	< 0.0001	33.0
	**BAI 5**[b]	-2.13	0.57	-3.75	100.0	0.0003	0.75	0.08	9.09	100.0	< 0.0001	9.1
	**BAI 10**[b]	-2.89	0.61	-4.69	100.0	< 0.0001	0.88	0.09	9.63	100.0	< 0.0001	3.7
	**Duct production 5**	2.61	0.28	9.28	5.4	0.0002	0.05	0.02	2.42	97.4	0.0172	54.4
	**Duct production 10**	2.33	0.27	8.49	7.8	< 0.0001	0.11	0.03	3.54	97.8	0.0006	48.3
	**Duct size 5**	2.25	0.34	6.61	14.6	< 0.0001	92.73	34.67	2.68	95.3	0.0088	53.0
	**Duct size 10**	2.30	0.37	6.26	20.4	< 0.0001	92.50	43.18	2.14	96.0	0.0347	55.5
	**Total duct area 5**	2.65	0.27	9.81	4.6	0.0003	6.02	2.18	2.77	96.6	0.0068	52.7
	**Total duct area 10**	2.38	0.25	9.49	6.0	< 0.0001	14.42	3.53	4.08	97.0	0.0001	44.7
	**Duct density 5**	3.09	0.23	13.41	3.7	0.0003	-0.28	0.14	-2.06	97.3	0.0425	55.9
	**Duct density 10**	3.20	0.23	13.75	4.3	0.0001	-0.51	0.21	-2.45	98.2	0.0159	54.4
	Relative duct area 5	2.97	0.25	11.77	3.9	0.0003	-0.07	0.09	-0.77	95.7	0.4427	59.3
	Relative duct area 10	3.01	0.27	11.31	4.7	0.0001	-0.12	0.15	-0.82	96.6	0.4123	59.3

All 100 trees characterized here are tamarack (*Larix laricina*) located in ELB impacted stands in Beltrami Island State Forest, Minnesota. [b] data are log transformed. Explanatory variables in bold typeface have slope p-values below a 0.05 threshold for statistical significance. DBH refers

to diameter at breast height and BAI refers to basal area increment. A full description of variables can be found in Table 2 of the primary manuscript.

Table S3: Relationships between phloem resin cell density and other tree characteristics in tamarack (*Larix laricina*) observed during an outbreak of eastern larch beetle (*Dendroctonus simplex*) based on linear mixed effects models.

Response Variable	Explanatory Variable	Intercept					Slope					
		EST	SE	*t*	*df*	*p*	EST	SE	*t*	*df*	*p*	ΔAIC
Phloem resin cell density[a]	.	2.74	0.25	11.08	2.6	0.0030	.	.	.	.	.	2.8
	**DBH**	3.74	0.42	8.84	26.7	< 0.0001	-0.06	0.02	-2.52	66.5	0.0141	0.0
	Age	3.69	0.57	6.52	54.1	< 0.0001	-0.02	0.01	-1.78	98.0	0.0776	1.9
	Stand density	2.67	0.29	9.16	4.7	0.0003	0.00	0.01	0.46	98.2	0.6476	4.6
	Ring width 5	2.55	0.28	9.22	4.5	0.0004	0.09	0.07	1.38	96.9	0.1703	2.9
	Ring width 10	2.47	0.29	8.50	5.5	0.0002	0.15	0.09	1.63	97.0	0.1068	2.2
	BAI 5[b]	2.51	0.86	2.93	87.5	0.0044	0.03	0.12	0.27	97.4	0.7850	4.7
	BAI 10[b]	2.46	0.96	2.58	90.4	0.0116	0.04	0.14	0.30	98.2	0.7671	4.7
	Duct production 5	2.71	0.29	9.45	4.8	0.0003	4.45 E-03	0.02	0.18	98.1	0.8561	4.7
	Duct production 10	2.69	0.32	8.44	6.9	< 0.0001	0.01	0.04	0.25	97.4	0.8060	4.7
	Duct size 5	2.81	0.39	7.24	15.4	< 0.0001	-9.56	41.94	-0.23	97.4	0.8200	4.7
	Duct size 10	2.72	0.43	6.39	20.4	< 0.0001	2.85	51.58	0.06	97.5	0.9560	4.8
	Total duct area 5	2.79	0.28	10.09	3.8	0.0007	-1.10	2.58	-0.43	97.4	0.6710	4.6
	Total duct area 10	2.76	0.30	9.35	5.2	0.0002	-0.65	4.30	-0.15	97.0	0.8803	4.7
	Duct density 5	2.69	0.26	10.35	3.4	0.0011	0.08	0.16	0.48	97.4	0.6328	4.5
	Duct density 10	2.75	0.28	9.88	4.1	0.0005	-0.02	0.24	-0.08	96.9	0.9401	4.8
	Relative duct area 5	2.72	0.26	10.46	3.2	0.0014	0.02	0.10	0.17	97.1	0.8623	4.7
	Relative duct area 10	2.77	0.28	9.95	4.1	0.0005	-0.04	0.17	-0.24	96.9	0.8106	4.7
	Phloem thickness	2.87	0.40	7.12	17.8	< 0.0001	-0.05	0.11	-0.42	98.2	0.6750	4.6

All 100 trees characterized here are tamarack (*Larix laricina*) located in ELB impacted stands in Beltrami Island State Forest, Minnesota. [a] data are square root transformed; [b] data are log transformed. Explanatory variables in bold typeface have slope p-values below a 0.05 threshold for statistical significance. DBH refers to diameter at breast height and BAI refers to basal area increment. A full description of variables can be found in Table 2 of the primary manuscript.

Table S4: Relationships between growth metrics and tree characteristics in tamarack (*Larix laricina*) observed during an outbreak of eastern larch beetle (*Dendroctonus simplex*) based on linear mixed effects models.

Response Variable	Explanatory Variable	Intercept					Slope					
		EST	SE	*t*	*df*	*p*	EST	SE	*t*	*df*	*p*	ΔAIC
DBH[a]	Intercept only	4.13	0.21	20.04	2.9	0.0003	.	.	.	.	.	20.5
	**Age**	3.23	0.38	8.54	21.4	< 0.0001	0.02	0.01	2.90	69.9	0.0050	17.4
	**Stand density**	4.44	0.23	19.24	3.6	0.0001	-0.02	3.74 E-03	-5.05	82.0	< 0.0001	0.0
Ring width 10[b]	Intercept only	0.42	0.15	2.80	3.0	0.07	.	.	.	.	.	24.5
	**DBH**	-0.59	0.25	-2.34	15.8	0.0325	0.06	0.01	5.30	53.2	< 0.0001	2.5
	**Age**	1.89	0.35	5.41	21.9	< 0.0001	-0.03	0.01	-4.31	27.3	0.0002	12.8
	**Stand density**	0.88	0.18	4.88	4.9	0.0048	-0.03	4.85 E-03	-5.45	128.6	< 0.0001	0.0
BAI 10[b]	Intercept only	6.75	0.21	31.56	4.2	< 0.0001	.	.	.	.	.	84.6
	**DBH**	4.79	0.22	21.88	11.6	< 0.0001	0.11	0.01	11.42	15.3	< 0.0001	0.0
	Age	7.60	0.48	15.78	24.6	< 0.0001	-0.02	0.01	-1.92	34.4	0.0634	83.9
	**Stand density**	7.33	0.25	29.39	6.2	< 0.0001	-0.03	0.01	-5.97	126.6	< 0.0001	55.5

All 129 trees characterized here are tamarack (*Larix laricina*) located in stands impacted by ELB in Beltrami Island State Forest, Minnesota. [a] data are square root transformed; [b] data are log transformed. Two outliers (see methods) were removed from the dataset to maintain equivalent sample size for AIC comparisons during model building. Explanatory variables in bold typeface have slope p-values below a 0.05 threshold for statistical significance. DBH refers to diameter at breast height and BAI refers to basal area increment. A full description of variables can be found in Table 2 of the primary manuscript.
